# Machine learning applications in vascular neuroimaging for the diagnosis and prognosis of cognitive impairment and dementia: a systematic review and meta-analysis

**DOI:** 10.1186/s13195-025-01815-6

**Published:** 2025-08-07

**Authors:** Valerie Lohner, Amanpreet Badhwar, Flavie E. Detcheverry, Cindy L. García, Helena M. Gellersen, Zahra Khodakarami, René Lattmann, Rui Li, Audrey Low, Claudia Mazo, Amelie Metz, Olivier Parent, Veronica Phillips, Usman Saeed, Sean Y. W. Tan, Stefano Tamburin, David J. Llewellyn, Timothy Rittman, Sheena Waters, Jose Bernal

**Affiliations:** 1 Cardiovascular Epidemiology of Aging, Department of Cardiology, Faculty of Medicine and University Hospital Cologne, University of Cologne, Kerpener Str. 62, 50937 Cologne, Germany; 2 Multiomics Investigation of Neurodegenerative Diseases (MIND) lab, 4545 Queen Mary Road, Montréal, QC H3W 1W6 Canada; 3 Department of Pharmacology and Physiology, Faculty of Medicine, Université de Montréal, 2900 boulevard Édouard-Montpetit, Montréal, QC H3T 1J4 Canada; 4 Institute of Biomedical Engineering, Université de Montréal, 2960 chemin de la Tour, Montréal, QC H3T 1J4 Canada; 5 Centre de Recherche de l’Institut Universitaire de Gériatrie de Montréal (CRIUGM), 4565 chemin Queen Mary, Montréal, QC H3W 1W5 Canada; 6 McGill University, 845 Sherbrooke St W, Montréal, QC H3A 0G4 Canada; 7 Douglas Research Centre, 6875 Boulevard LaSalle, Montréal, QC H4H 1R3 Canada; 8 German Centre for Neurodegenerative Diseases (DZNE), Leipziger Str. 44/Haus 64, 39120 Magdeburg, Germany; 9 MRC Cognition and Brain Sciences Unit, University of Cambridge, 15 Chaucer Rd, Cambridge, CB2 7EF UK; 10 Department of Psychology, University of Cambridge, Downing Pl, Cambridge, CB2 3EB UK; 11 Department of Bioengineering, School of Engineering and Applied Science, University of Pennsylvania, 240 Skirkanich Hall, 210 S 33rd St, Philadelphia, PA 19104 USA; 12 Penn Image Computing and Science Laboratory (PICSL), Department of Radiology, University of Pennsylvania, 3400 Spruce Street 1 Silverstein Philadelphia, Philadelphia, PA 19103 USA; 13 Institute of Cognitive Neurology and Dementia Research (IKND), Otto-von-Guericke University, Leipziger Str. 44/Haus 64, 39120 Magdeburg, Germany; 14 Department of Clinical Neurosciences, University of Cambridge, Hills Road, Cambridge, CB2 0XY UK; 15 Department of Psychiatry, University of Cambridge, Robinson Way, Cambridge, CB2 0SZ UK; 16 Dublin City University, Collins Ave Ext, Whitehall, Dublin 9, Dublin, Ireland; 17 University of Cambridge Medical Library, Hills Rd, Cambridge, CB2 0SP UK; 18 Institute of Medical Science, Temerty Faculty of Medicine, University of Toronto, 1 King’s College Circle, Toronto, ON M5S 1A8 Canada; 19 Hurvitz Brain Sciences Program, Sunnybrook Research Institute, 2075 Bayview Avenue, Toronto, ON M4N 3M5 Canada; 20 Department of Neurosciences, Biomedicine and Movement Sciences, University of Verona, Piazzale Ludovico Antonio Scuro 10, 37124 Verona, Italy; 21 University of Exeter, Stocker Rd, Exeter, EX4 4PY UK; 22 Queen Mary University of London, Mile End Road, London, E1 4NS UK; 23 Centre for Clinical Brain Sciences (CCBS), Department of Neuroimaging Sciences, The University of Edinburgh, 49 Little France Crescent, Edinburgh, EH16 4SB UK

**Keywords:** Machine learning, Dementia, Cerebral small vessel disease, Artificial intelligence, Neuroimaging, Cognitive impairment, Alzheimer’s dementia, Neurodegenerative diseases

## Abstract

**Background:**

Cerebral small vessel disease (CSVD) is a common neurological condition that contributes to strokes, dementia, disability, and mortality worldwide. We conducted a systematic review and meta-analysis to investigate the use of neuroimaging CSVD markers in machine learning (ML) based diagnosis and prognosis of cognitive impairment and dementia, and identify both methodological changes over time and barriers to clinical translation.

**Methods:**

Following the PRISMA guidelines, we systematically searched for original studies that used both neuroimaging CSVD markers and ML methods for diagnosing and prognosing neurodegenerative diseases (preregistration in PROSPERO: CRD42022366767). Each paper was independently reviewed by a pair of reviewers at all stages, with a third consulted to resolve conflicts. We meta-analysed the effectiveness of ML models to distinguish healthy controls from Alzheimer’s dementia and cognitive impairment, using area under the curve (AUC) as the performance metric.

**Results:**

We identified 75 studies: 43 on diagnosis, 27 on prognosis, and 5 on both. Nearly 60% of studies were published in the past two years, reflecting a growing interest in using CSVD markers in ML-based diagnosis and prognosis of neurodegenerative diseases, especially Alzheimer’s dementia. This rising interest may be linked to the strong performance of such models: according to our meta-analysis, ML approaches using CSVD markers perform well in differentiating healthy controls from Alzheimer’s dementia (AUC 0.88 [95%-CI 0.85–0.92]) and cognitive impairment (AUC 0.84 [95%-CI 0.74–0.95]). However, the growing interest has not been matched by methodological rigour: only 16 studies met the criteria for inclusion in the meta-analysis due to inconsistent reporting, only five assessed the generalisability of their models on external datasets, and six lacked clear diagnostic criteria.

**Conclusions:**

Interest in incorporating CSVD markers into ML models for neurodegenerative disease classification is on the rise, and their performance suggests that this is worth further exploration. Serious methodological issues, including inconsistent reporting, limited generalisability testing, and other potential biases, are unfortunately common and hinder further adoption. Our targeted recommendations provide a roadmap to accelerate the integration of ML into clinical practice.

**Supplementary Information:**

The online version contains supplementary material available at 10.1186/s13195-025-01815-6.

## Introduction

Cerebral small vessel disease (CSVD) describes multiple dynamic pathological processes that impair the optimal functioning of perforating arterioles, capillaries, and venules in the brain [1–3]. CSVD is among the most common conditions encountered by neurologists in clinical practice [4] and a significant contributor to major healthcare challenges. CSVD causes 25% of ischaemic strokes, the majority of intracerebral haemorrhages in individuals over 65 years old, and most cases of vascular dementia [2, 3]; contributes to around 45% of all dementia cases worldwide [4]; and leads to mobility and gait issues, neurobehavioural changes, and mood disorders [5]. The relationship between CSVD and Alzheimer’s disease (AD) has been recognised since the earliest days of AD research [6], and is now included in the Alzheimer’s Society’s most recent revised criteria for diagnosing and staging AD [7]. This coexistence between CSVD and AD has taken on a new significance in recent years, as anti-amyloid monoclonal antibody trials have revealed that individuals with cerebral amyloid angiopathy — a form of CSVD — are at risk of developing brain swelling or haemorrhages during the course of the treatment, making CSVD assessments and studies crucial for patient stratification and minimising treatment risks [8].

Although direct assessment of the human cerebral microvasculature in vivo remains challenging with standard imaging technologies, its chronic dysfunction leads to changes that can be detected through magnetic resonance imaging (MRI) and computed tomography (Supplementary BOX 1). Assessing CSVD has traditionally focused on evaluating discrete lesions, such as white matter hyperintensities (WMH), lacunes, cerebral microbleeds, superficial siderosis, perivascular spaces, and small subcortical or cortical microinfarcts, by means of clinical visual ratings and increasingly through quantitative methods [1]. However, advancements in neuroimaging technologies have also revealed that these discrete lesions are not the only consequence, suggesting instead that they often lead to widespread, rather than focal, alterations of microstructure and connectivity [9].

The integration of neuroimaging and machine learning (ML) techniques (Supplementary BOX 2) presents new avenues for understanding the intricate and multifactorial nature of CSVD and vascular contributors to cognitive impairment and dementia [10]. These possibilities include not only the computational quantification of neuroimaging markers of CSVD (e.g., through segmentation of lesions) [11–13] but also their predictive value for neurodegenerative diseases and dementia, which could ultimately facilitate early detection and personalised treatments. However, the contribution of CSVD to dementia and cognitive impairment using ML appears to be underdeveloped. According to a recent systematic review and meta-analysis conducted by the Imaging Working Group of the international Deep Dementia Phenotyping Network (DEMON) on the application of neuroimaging and ML for dementia diagnosis and prognosis [14], only 2 out of 255 studies focused on vascular forms of dementia. Whilst that review did not consider cognitive changes other than dementia and most included studies leveraged the Alzheimer’s Disease Neuroimaging Initiative (ADNI)—a cohort with relatively minimal CSVD burden—it is surprising so few studies had been conducted in this field.

To map the significance of CSVD in ML-based detection of dementia and cognitive impairment more generally, we established a new subgroup of the DEMON Imaging Working group dedicated specifically to this topic. We conducted a systematic review and meta-analysis to (a) determine the use of CSVD neuroimaging markers and ML in the diagnosis and prognosis of cognitive impairment and dementia; (b) identify methodological shifts over time, particularly with recent deep learning advancements; and (c) pinpoint methodological barriers preventing the development and effective deployment of these strategies. Our primary focus was on papers addressing dementia-related diagnosis and prognosis rather than those solely centred on lesion segmentation. We aim for this review to inspire the development of more accurate and validated methods for predicting CSVD-related cognitive impairment, facilitating early detection and intervention.

## Methods

### Protocol registration

We registered this systematic review protocol with the International Prospective Register of Systematic Reviews (PROSPERO), registration number: CRD42022366767. We conducted this work following the Preferred Reporting Items for Systematic Reviews and Meta-Analysis (PRISMA) Statement [15]. The associated PRISMA checklist can be found in the supplementary material.

### Search strategy

A medical librarian (VP) searched databases Medline (via Ovid), Embase (via Ovid), Cochrane Library, Emcare (via Ovid), Cinahl (via Ebscohost), PsycInfo (via Ebscohost), BNI (via ProQuest), Web of Science (Core Collection), and Scopus from inception to the date searches were conducted. The search strategy was peer-reviewed using the Peer Review of Electronic Search Strategies (PRESS) checklist [16], and evaluated against the PRISMA-S guidelines [17]. Databases were searched separately, rather than multiple databases being searched on the same platform. The search syntax was adapted for each database, and to account for variation between thesaurus terms/controlled vocabulary across each database. Results were limited to the English language in all databases. Results were exported to Endnote 20 for deduplication, using the method outlined by Bramer et al. [18].

All searches were originally conducted on September 20, 2023 and rerun on September 9, 2024 to include any papers published between the initial search and final submission.

### PICOS framework

The parameters of this systematic review, as defined by the PICOS framework, were as follows:

- *P*articipants: Persons with cognitive impairment or a clinical diagnosis of dementia, as well as people with incident cognitive impairment or dementia.
- *I*ndex: Neuroimaging-derived CSVD data analysed with ML for diagnosis or prognosis.
- *C*omparator:
  - For diagnostic studies: persons without cognitive impairment or dementia.
  - For prognostic studies: prognostic factor (conversion to cognitive impairment or dementia vs. no conversion).
- *O*utcome: Accuracy of diagnosis or prognosis of cognitive impairment or dementia based on CSVD burden.
- *S*tudy design: Original cross-sectional or prospective observational studies.

### Inclusion and exclusion criteria

To be included, studies had to report on the model performance of the ML methods for the diagnosis or prognosis of cognitive impairment or dementia using imaging markers of CSVD and ML. We deemed eligible original studies published in English in peer-reviewed journals and excluded in vitro studies or animal studies. We also excluded studies that employed ML solely for image processing such segmentation.

### Study selection

Study selection had two stages. First, each report was screened for eligibility by pairs of independent reviewers based on title and abstract using the screening tool Rayyan (https://www.rayyan.ai/). Second, each report that passed the initial filtering was reviewed by pairs of reviewers who independently conducted full-text screening. Conflicts arising at any of these two stages were resolved through discussions, with the assistance of a third independent senior reviewer when necessary.

### Data extraction

Data from each included study were extracted independently by pairs of reviewers using a standard template. Once again, conflicts were resolved through discussion, with a third senior reviewer solving any remaining disagreements. The data extraction form captured (a) article information (first/last author, year, journal, country of first/last author’s affiliated institution, study type); (b) characteristics of the study population (sample size, age, sex, race/ethnicity, criteria for cognitive impairment and dementia, inclusion and exclusion criteria of study); (c) data analysis (ML approach used, covariates included in model, vascular neuroimaging features used; outcome measure); (d) results (measures of model performance (accuracy, sensitivity, specificity, area under the curve (AUC), positive predictive value, negative predictive value), other metrics reported (e.g. hazard or odds ratios), follow-up period (for prognostic studies only); and (e) risk of bias assessment.

### Assessment of risk of bias

We assessed the quality of all individual studies using the QUality Assessment of Diagnostic Accuracy Studies (QUADAS-2) for diagnostic studies [19] and the Prediction model Risk Of Bias ASsessment Tool (PROBAST) for prognostic studies [20]. Pairs of reviewers independently conducted the critical appraisal of each paper and certainty of evidence rating. Disagreements were resolved through discussion.

### Meta-analysis

We conducted a meta-analysis to provide a targeted evaluation of the ML models using vascular neuroimaging features. This meta-analysis focused on the two most common diagnostic tasks identified in the literature we reviewed: distinguishing between healthy controls and AD-dementia or all-cause dementia, as well as between healthy controls and cognitive impairment. Thus, we compared the performance of the various approaches using the AUC obtained from Receiver Operating Characteristic analyses.

We employed a random-effects model with the DerSimonian and Laird estimation method to calculate the pooled AUC values and confidence intervals. In cases of missing data, such as absent variability measures for the AUC, we reached out to the corresponding authors. In instances where authors did not respond to our inquiries and studies failed to report any measures of variability for the AUC, we estimated the standard error for the AUC based on Hanley and McNeil [21]. If different ML approaches were considered for the same database, we included the analysis utilising the ML model with the best model fit and the largest sample size. We quantified heterogeneity using Cohen’s Q statistics and I^2^ statistics. The meta-analysis was performed in R version 4.2.1 [22] using the package *metafor* [23].

## Results

### Search results

**Fig. 1:**
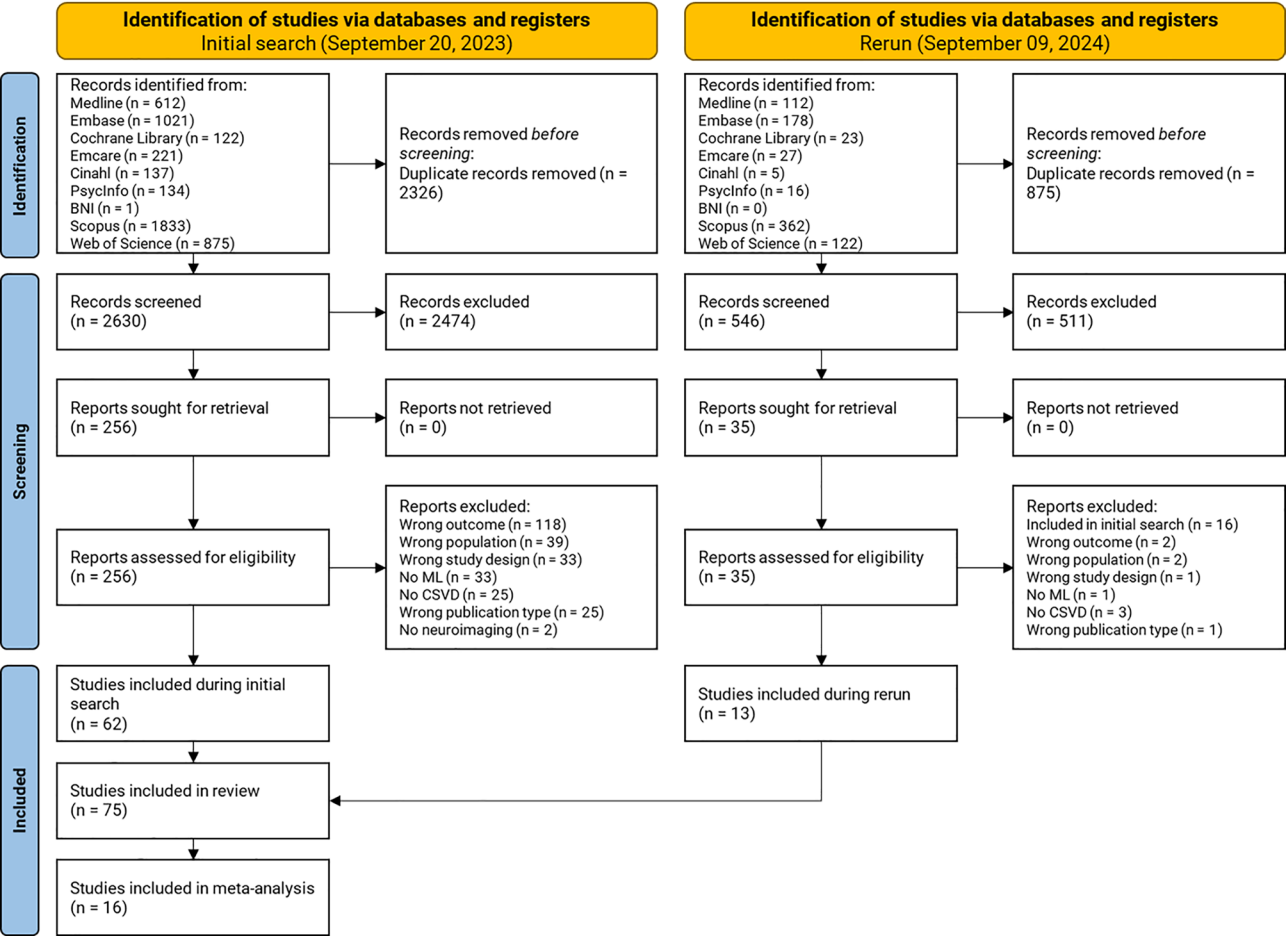
PRISMA flow chart outlining the number of studies identified, included, and excluded at each stage of the systematic review and meta-analysis

Our initial search on September 20, 2023, identified 4,956 potentially relevant records across all databases (Fig. 1). After deduplication using Endnote and Rayyan, we retained 2,630 records. Of these, 256 passed the title and abstract screening, and 62 went on to pass full-text screening and were included in the systematic review [24–85]. On September 9, 2024, we conducted a rerun and identified 845 potentially new relevant records across the same databases (Fig. 1). A total of 546 records remained after deduplication. Screening by title and abstract led to 35 eligible papers, of which 13 passed full-text screening and were included in the systematic review [86–98]. This brought the total number of included records to 75 [24–98]. We contacted the corresponding authors of 36 studies via email to request missing data. Of these, six responded: three provided additional data, while the other three were unable to do so. Finally, a total of 16 studies was suitable for meta-analysis [30, 32, 33, 47, 51, 58, 65, 79, 84, 85, 89, 91, 96, 98], of which seven classified healthy controls versus AD-dementia [30, 32, 47, 58, 65, 84, 89], and nine healthy controls versus cognitive impairment [32, 33, 51, 58, 79, 85, 91, 96, 98]. A flow chart of the identification and screening process is provided in Fig. 1. Data extraction results can be found in Supplementary data 2.

### Study characteristics

Comprehensive details on data extraction related to study characteristics are available in Supplementary Data 2, under the sections “description / metrics paper” and “general description of the study population”.

#### Origin of studies

**Fig. 2:**
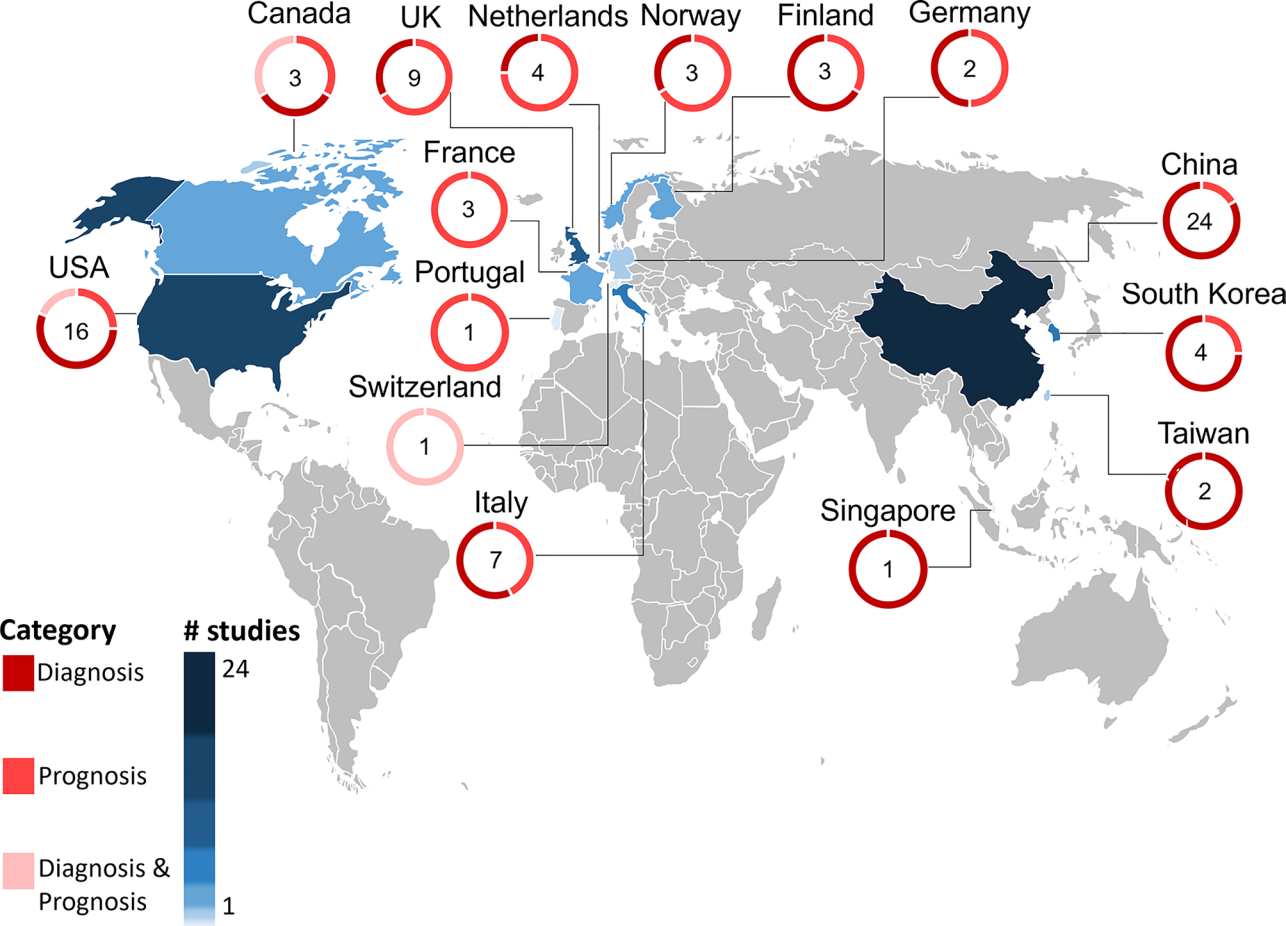
Countries of institutional affiliation for the first and last authors of each included publication. Note that the first and last authors had different affiliations in eight papers. The numbers on the map thus sum to 83 rather than 75 (total number of included studies)

According to the affiliations of the first and last authors, the majority of included studies were from China (*n* = 24) and the United States of America (USA) (*n* = 16). The remaining studies originated primarily from Europe (*n* = 33), followed by Asia (*n* = 7, excluding China), and North America (*n* = 3, excluding the USA). No studies were affiliated with institutions in South America, Africa, or Australia (Fig. 2).

#### Study focus

Among the 75 included studies, 43 (57%) focused on diagnosis, 27 (36%) on prognosis, and five (7%) on both (Supplementary Table 1). More than two-thirds (70%) of the studies used private or local datasets (*n* = 35 diagnosis, *n* = 16 prognosis, *n* = 2 both). When publicly available datasets were used (*n* = 12 for diagnosis, *n* = 14 for prognosis, *n* = 4 for both), ADNI was the most frequent choice (*n* = 17/30). Only 14 studies included two or more cohorts in their analyses (*n* = 7 for diagnosis, *n* = 5 for prognosis, *n* = 2 for both). ADNI was the most commonly used dataset in these cases (*n* = 9/14).

#### Participant demographics

The mean age of participants was 71.7 years (standard deviation (SD) 8.5), with a mean age of 69.9 years (SD 9.3) in diagnostic studies and 72.1 years (SD 8.3) at baseline in prognostic studies. There was a relatively balanced representation of men and women across the studies, with women making up 54% of participants overall (48.1% in diagnostic studies and 55% in prognostic studies).

Only six studies (8%) provided race or ethnicity information (*n* = 3 diagnosis, *n* = 3 prognosis). In those studies, 6217 (79%) participants were reported as White (*n* = 1 diagnosis, *n* = 3 prognosis), 209 (3%) as Asian (*n* = 2 diagnosis), 934 (12%) as Black (*n* = 1 prognosis), and 476 (6%) as “other” (*n* = 2 diagnosis, *n* = 2 prognosis).

### ML methods

Detailed information on the ML methods used in each study can be retrieved from Supplementary Data 2, specifically from the section “ML method used”.

#### Application of ML methods

**Fig. 3:**
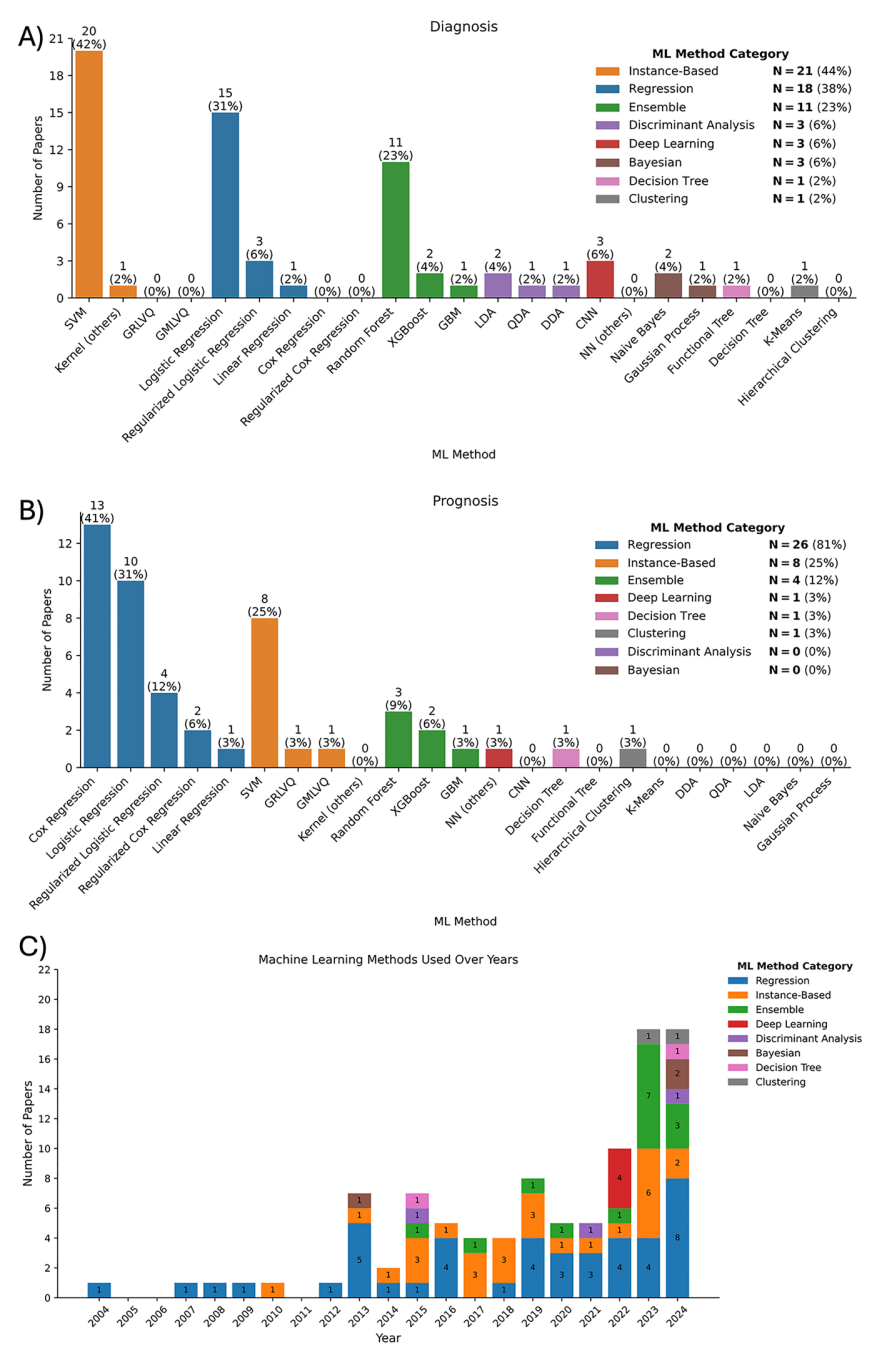
Number of papers using each machine learning (ML) method in our review, faceted by **A**) diagnosis or **B**) prognosis, and **C**) use of different ML categories over time. Different colours correspond to different ML categories. Note that as one paper may use multiple ML methods/categories, the percentages in **A**) and **B**) may not sum up to 100%. Kernel (others) = Kernel methods other than SVM; NN (others) = Neural networks other than CNN

A total of 23 different ML methods were employed for the diagnosis or prognosis of cognitive impairment and dementia based on vascular neuroimaging features (Fig. 3). These methods spanned eight categories (Supplementary BOX 2). For diagnosis (Fig. 3A), the most popular ML categories were instance-based, regression, and ensemble algorithms, with support vector machines (SVM, instance-based), logistic regression (regression), and random forest (ensemble) being the most commonly used models. For prognosis (Fig. 3B), the top three categories remained the same, with Cox regression (regression), SVM (instance-based), and random forest (ensemble) being the most frequently used models.

Logistic regression, was the most popular ML method, appearing in 28 (37%) of the 75 papers reviewed [24, 27, 41, 44, 50, 53, 54, 58–60, 63, 64, 66, 67, 72, 74–76, 81–83, 86, 87, 91, 93, 94, 96, 98]. It was followed closely by SVM which was used in 26 (35%) papers [25, 28, 33, 35–40, 45, 46, 48–52, 72, 79, 82, 84, 85, 90, 93, 94, 97, 98]. These two methods were applied almost twice as often as the next most common methods, namely Cox regression and random forest, which were featured in 15 (20%) [42, 55, 62, 68–70, 73, 75, 77, 78, 80, 88, 89, 92, 99] and 14 (19%) papers [32, 34, 50, 56, 57, 61, 72, 76, 82, 89, 91, 93, 97, 98], respectively. Other methods were much less common. Strikingly, despite the growing prominence of deep learning over the past decade, its application in diagnosing and predicting cognitive impairment and dementia based on vascular neuroimaging features remains limited. Only four (5%) of the papers employed deep learning [29, 30, 43, 47], with three of them using convolutional neural networks (CNNs) [30, 43, 47]. Three studies employed Bayesian methods [39, 91, 98] and three discriminant analysis [45, 71, 91], all exclusively applied for diagnostic purposes.

#### Popularity over time

The application of ML techniques for diagnosis and prediction of cognitive impairment and dementia based on vascular neuroimaging features has experienced a significant growth over the past decade (Fig. 3C). Cox regression was the first technique we identified that used any CSVD feature, namely WMH, to predict dementia [99]. Logistic regression and SVM have remained popular and widely used since their first application in 2007 and 2010, respectively. The application of random forests, on the other hand, was first identified in 2015 and has only recently reached its highest level of usage, as it was used in around one-third of the works in 2023 (36%). Neural networks and eXtreme Gradient Boosting (XGBoost) appeared only in 2022.

#### Validation and generalisation

A rigorous evaluation of both performance and generalisability of ML prediction algorithms is essential for the translation into real-world clinical settings, as even the most promising models may fail when exposed to previously unseen data. The authors’ approaches to validation are outlined in Supplementary Data 2, under the section “Validation strategy”. A representative illustration of the importance of using an independent hold-out test set is provided in a paper included in this review [91], where the authors showed that the model with the highest performance on the training set al.so exhibited the poorest generalisation, with an AUC of 100 [95% CI: 100, 100] in training and 50.0 [95% CI: 50.0, 50.0] in an independent test set. Despite this, only five out of 75 studies assessed the generalisation capabilities of their models using held-out external datasets (*n* = 4 diagnosis; *n* = 1 prognosis) [47, 48, 61, 88, 91]. Although limited access to multiple datasets clearly presents a challenge for assessing generalisability, a total of ten studies had data from multiple sources and were therefore well-positioned to validate their models on independent cohorts (*n* = 5 diagnosis [31, 47, 48, 90, 91]; *n* = 3 prognosis [88, 93, 94]; *n* = 2 both [76, 89]).

When reported clearly, studies relied to a large extent on cross-validation (*n* = 28 diagnosis; *n* = 10 prognosis). Some studies also split a single or pooled dataset into training and testing sets from the start (*n* = 3 diagnosis; *n* = 3 prognosis) [43, 47, 48, 61, 69, 88, 91] and one diagnostic study also used bootstrapping [71]. Unfortunately, the absence of any form of validation—or a lack of clear reporting on whether it was conducted—was strikingly common, especially in prognostic analyses, where over 55% of studies were affected by this issue (*n* = 12 diagnosis; *n* = 18 prognosis).

### Neuroimaging modalities and features

**Fig. 4:**
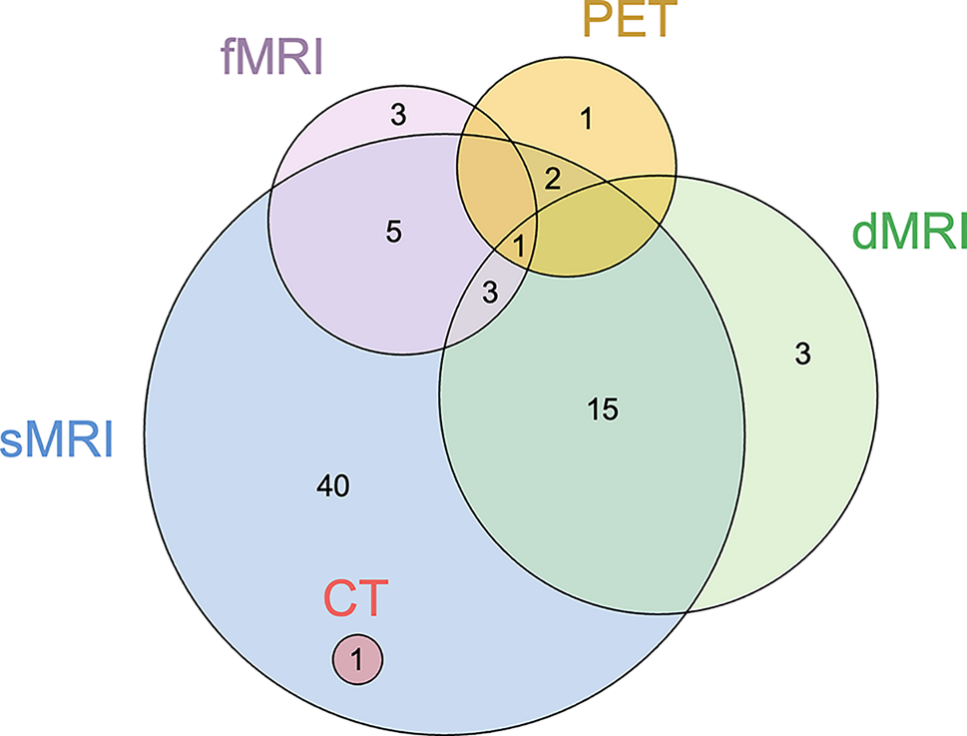
Neuroimaging techniques used to extract vascular neuroimaging features in the studies included in the review. Abbreviations: CT = computed tomography; dMRI = diffusion-based MRI; fMRI = functional MRI, sMRI = structural MRI; PET = positron emission tomography

Data extracted regarding neuroimaging modalities and features can be found in Supplementary Data 2, under the sections “specific details for datasets” and “vascular neuroimaging features used”. Studies leveraged structural, diffusion, and functional MRI (fMRI), as well as computed tomography and positron emission tomography (Fig. 4). Structural MRI was by far the most widely used neuroimaging modality, appearing in 67 (89%) studies, whereas computed tomography was the least used, appearing in only one (1%) paper. A total of 27 (36%) studies leveraged two or more imaging modalities. When this occurred, structural MRI and diffusion-based MRI were the most common combination, used in 19 out of 27 (70%) papers.

#### Diagnosis

Around 40% of the diagnostic studies (*n* = 20) leveraged vascular neuroimaging features obtained from structural MRI sequences. Every study that used structural MRI quantified WMH. Other neuroimaging features were less frequently considered: perivascular spaces (*n* = 2) [41, 82], cerebral microbleeds (*n* = 2) [41, 82], lacunes (*n* = 3) [41, 81, 82], and stroke lesion volume or subtype (*n* = 2) [54, 67].

Non-lesion measurements were also common in many diagnostic studies. These included assessments of microstructural integrity from regional white matter (*n* = 13) [32, 34, 36–39, 51, 54, 61, 65, 71, 79, 83], fMRI or diffusion MRI-based connectivity (*n* = 8) [33, 35, 45, 49, 51, 76, 84, 85], or other, less conventional imaging features (*n* = 7), such as tissue textures (*n* = 2) [56, 89], white matter density (*n* = 1) [65], fMRI-derived amplitude of low-frequency fluctuation (*n* = 3) [53, 61, 96], ¹⁸F-fluorodeoxyglucosepositron emission tomography derived “metabolic cognitive signature” (*n* = 1) [47], and iron deposition (*n* = 1) [40].

Of the 48 studies reporting diagnostic analyses, most used quantitative assessments of vascular neuroimaging features (*n* = 40). Nine (19%) employed clinical visual ratings, of which two relied solely on visual ratings.

#### Prognosis

Twenty-four (75%) prognostic studies used vascular neuroimaging features derived from structural MRI. WMH were assessed in every study. Other neuroimaging features, including lacunes (*n* = 7) [42, 46, 55, 63, 80, 94, 95], perivascular spaces (*n* = 5) [42, 80, 86, 92, 95], cerebral microbleeds (*n* = 5) [42, 72, 92, 94, 95], and stroke lesion volume or subtype (*n* = 2) [24, 25], were less relatively common.

Non-lesion measurements were less common in prognostic than in diagnostic studies. Five studies used diffusion tensor imaging [29, 62, 78, 88, 94] and no studies investigated other diffusion-based signal modelling. Two studies investigated connectivity measures: one using diffusion-based structural connectivity [76], and one using WMH-based disconnectome measures [50]. Other vascular markers used in prognosis studies included tissue texture analysis (*n* = 2) [69, 70], and susceptibility-weighted imaging-based markers such as iron deposition (*n* = 1) [40].

Visual ratings were used in half of studies reporting prognostic results (16 out of 32). Most ratings were conducted on WMH burden, with one study reporting solely perivascular space ratings. Nine studies included additional quantitative measures alongside visual ratings, while the remaining relied solely on visual ratings (*n* = 7). There was significant variation in the rating scales used across studies. For example, the six prognostic publications that used visual ratings of WMH employed a range of methods, including binary measures with different cut-offs as well as grading scales with 3, 4, 10, and 11 points.

#### Temporal changes in vascular neuroimaging features

Studies using diffusion-based indices (e.g., fractional anisotropy and mean diffusivity) have decreased in recent years. Before 2020, diffusion imaging studies made up 41% of studies (*n* = 16), compared to just 17% of studies published between 2020 and 2024 (*n* = 6). Recent years have witnessed the adoption of novel techniques including graph theory, pattern analyses, and other methods of connectivity assessment, amplitude of low-frequency fluctuation, and composite brain signatures, making up a fifth (19%; *n* = 7) of studies published in 2020–2024. Studies relying solely on clinical visual ratings have not changed in recent years (*n* = 5 before 2020, *n* = 4 since 2020).

#### MRI scanner strength

MRI scanner strength details were reported in 65 (84%) studies (*n* = 40 diagnostic, *n* = 26 prognostic, *n* = 5 both). Diagnostic studies were primarily carried out on 3 Tesla (T) scanners. Most studies used a consistent scanner strength across participants and scans (*n* = 33), with 3T being the most common (*n* = 27), followed by 1.5T (*n* = 5), and one study using a 4T scanner. A few studies (*n* = 7) used scanners of different field strengths; six of these combined 1.5T and 3T scanners, while one study used both 1T and 1.5T scanners.

Most prognostic studies, on the other hand, were conducted on 1.5T scanners. Among the 27 prognostic studies that reported scanner strength, the majority used only 1.5T scanners (*n* = 15), with about half as many relying exclusively on 3T scanners (*n* = 7). A few studies (*n* = 5) employed a mix of scanner strengths; two combined 0.5T and 1.5T scanners, and three studies used both 1.5T and 3T scanners.

The use of higher field strength scanners has increased over time. Before 2020, more than half of the studies (59%) relied predominantly on 1.5T scanners (*n* = 23). Since 2020, reliance on 1.5T scanners has declined, with only 14% of studies using 1.5T scanners (*n* = 4), while 62% (*n* = 18) used 3T scanners exclusively.

#### Harmonisation

Since data acquisition often takes place across multiple sites using potentially different MRI scanners and protocols, harmonisation becomes crucial to deal with inter-scanner variability and ensure comparability across sites. We identified 19 studies that, in principle, would have benefited from harmonisation due to the use of different MRI scanners [25, 27, 32, 34, 39, 42, 48, 56–58, 69, 73, 77, 86, 88–91, 93]. Among these, five studies reported implementing any form of harmonisation: intensity normalisation across scanners [32], principal component analysis to isolate disease-relevant principal components while reducing scanner effects [39], manual correction of segmentation maps to mitigate inter-scanner variability [56, 57], and statistical harmonisation using ComBat [90].

### Dementia

Extracted data relevant to dementia diagnosis and prognosis are available in Supplementary Data 2, under the sections “diagnosis or prognosis” and “general description of the study population”. We identified 47 studies that focused on dementia, with 26 (55%) targeting diagnosis, 17 (36%) prognosis, and four (9%) both. Remarkably, despite the review’s strong emphasis on vascular aspects, diagnostic studies predominantly concentrated on AD (*n* = 24) [27, 28, 30, 32, 34, 36, 39, 47, 48, 52, 56–58, 60, 65, 66, 71, 74, 76, 82, 84, 89, 90, 97]. Other forms of dementia were also examined but with less frequency: vascular dementia (*n* = 4) [43, 45, 66, 82], frontotemporal dementia (*n* = 3) [82, 83, 90], Lewy body dementia (*n* = 3) [56, 57, 90], behavioural variant frontotemporal dementia (*n* = 1) [83], post-stroke dementia (*n* = 1) [47], progressive non-fluent aphasia (*n* = 1) [83], and semantic dementia (*n* = 1) [83]. Prognostic studies also focused largely on AD (*n* = 13) [26, 50, 52, 55, 59, 60, 63, 68–70, 76, 89, 94], with vascular dementia following (*n* = 6) [26, 46, 55, 73, 78, 94]. Mixed dementias were each examined by four prognostic studies [59, 63, 73, 75], and a single prognostic study also explored frontotemporal dementia [73].

#### Assessment criteria

Dementia diagnosis was primarily relied on published clinical criteria or via consensus diagnosis by experienced neurologists based on clinical evaluations, including cognitive tests, general neurological exams, and collateral information. Studies of AD employed no less than six different diagnostic criteria, namely Diagnostic and Statistical Manual of Mental Disorders (DSM versions III-R, IV or V) [100], National Institute of Neurological and Communicative Disorders and Stroke and the Alzheimer’s Disease and Related Disorders Association (NINCDS or NINCDS-ADRDA) [101], National Institute on Aging and Alzheimer’s Association (NIA-AA) [102, 103], National Institute on Aging–Alzheimer’s Disease Centers (NIA-ADRC), National Institute of Neurological Disorders and Stroke and Association Internationale pour la Recherche et l’Enseignement en Neurosciences (NINDS-AIREN) [104], and Alzheimer’s Disease Diagnostic and Treatment Centers (ADDTC) [105].

In addition to the diagnostic variability, six studies did not specify the criteria for diagnosing dementia (*n* = 4 diagnosis, *n* = 2 prognosis) and six diagnostic studies determined dementia diagnoses without adhering to standard criteria. Instead, they relied on specific thresholds from cognitive tests, including the Mini-Mental State Examination (MMSE) (*n* = 1), the Clinical Dementia Rating scale (CDR) (*n* = 1), the Montreal Cognitive Assessment (MoCA) scores (*n* = 1), and various combinations of these test (*n* = 3).

### Cognitive impairment

Information related to the diagnosis and prognosis of cognitive impairment, as extracted from the included studies, is provided in Supplementary Data 2 under the sections “diagnosis or prognosis” and “general description of the study population”. We identified 45 articles addressing cognitive impairment (without a specific dementia diagnosis), with 26 (58%) focusing on diagnosis, 14 (31%) on prognosis, and five (11%) assessing both diagnosis and prognosis. The definition, subtype, and potential aetiology of cognitive impairment varied substantially across the studies.

Most diagnostic studies (52%) that studied cognitive impairment examined cognitive impairment linked to CSVD (*n* = 11) [32, 33, 35, 38, 53, 54, 61, 71, 81, 91, 96], such as WMH (*n* = 4) [33, 35, 85, 91], subcortical ischemic vascular disease (*n* = 4) [32, 53, 71, 81], and vascular cognitive impairment (*n* = 3) [38, 54, 81]. However, a notable proportion of them (62%) also targeted mild cognitive impairment (MCI) (*n* = 12) [32, 37, 40, 41, 45, 49, 51, 58, 76, 87, 89, 98] or its amnestic and non-amnestic subtypes (*n* = 3) [27, 34, 79]. Cognitive impairment associated with Parkinson’s disease (*n* = 1) [44] and coronary artery disease (*n* = 1) [51] was also examined. Prognostic studies mainly investigated either the progression of MCI to dementia (*n* = 14) [40, 50, 52, 55, 59, 60, 69, 73, 76, 86, 89, 92, 93, 95], including progression to vascular dementia (*n* = 1) [73], mixed dementia (*n* = 1) [73], and AD (*n* = 6) [50, 52, 60, 69, 76, 86].

#### Assessment criteria

While there was some consistency across studies, the diagnostic criteria consisted of a variety of definitions of cognitive impairment. Most papers that focused on cognitive impairment relied on neuropsychological tests (*n* = 18 diagnosis, *n* = 11 prognosis. For example, performance below 1.5 standard deviations from the mean on cognitive tests was deemed indicative of MCI. A significant variability in the choice of cognitive tests was noted. Some papers adopted established clinical criteria, such as those proposed by Petersen et al. [106, 107] or the DSM-5 [100], or utilised consensus diagnoses from multiple neurologists (*n* = 1 diagnosis, *n* = 4 prognosis). In the other instances, a combination of neuropsychological tests and clinical criteria was used (*n* = 5 diagnosis, *n* = 2 prognosis) with some studies also incorporating vascular neuroimaging measures, such as including WMH burden to support MCI diagnosis (*n* = 5 diagnosis, *n* = 1 prognosis). Three papers did not specify the criteria used to identify MCI.

### Results meta-analysis

#### Healthy controls versus Alzheimer’s dementia

**Fig. 5:**
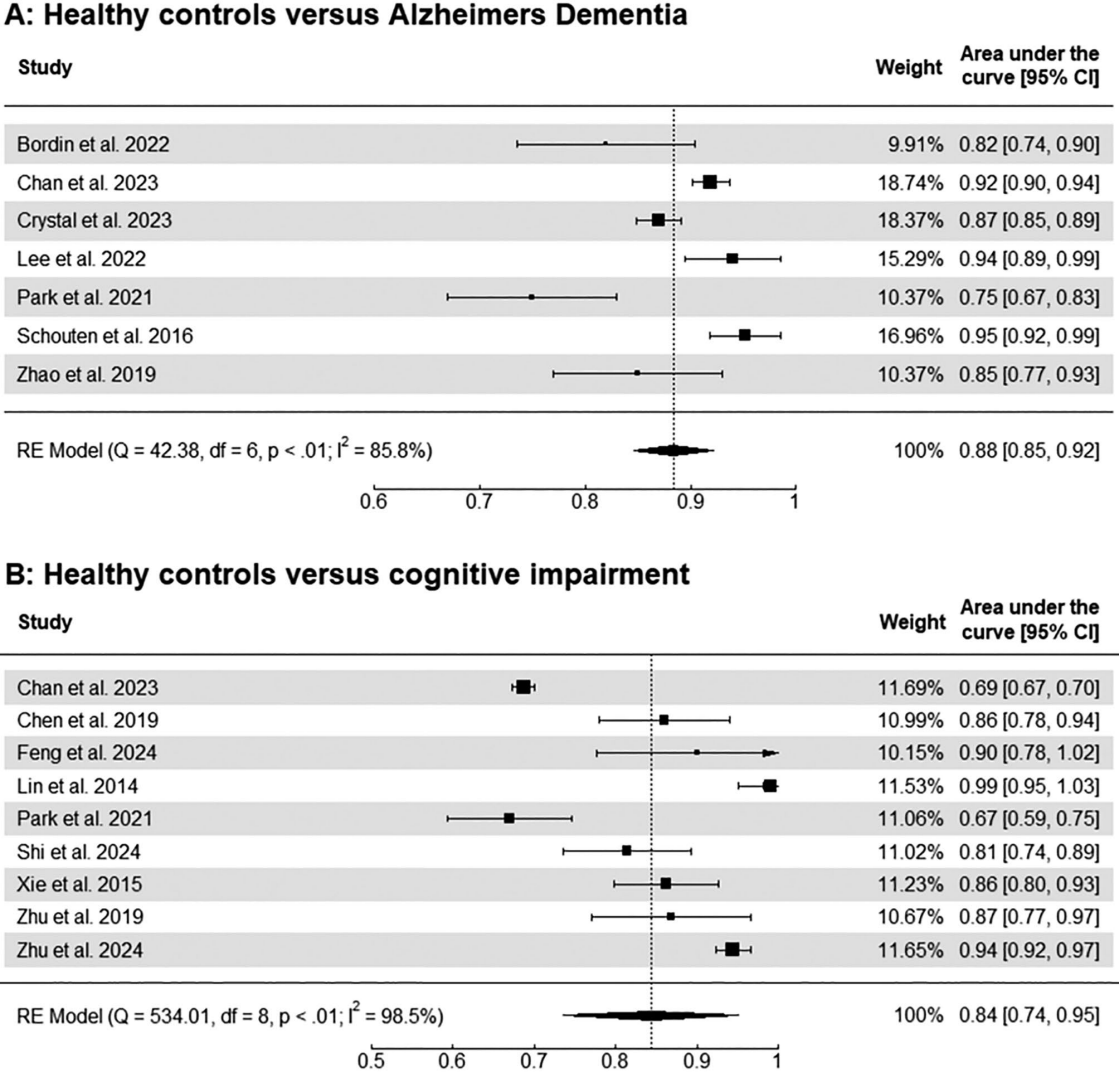
Meta-analysis of studies targeting the classification of (**A**) Alzheimer’s dementia versus healthy controls and (**B)** cognitive impairment versus healthy controls. Pooled AUC values and their corresponding confidence intervals (CI) were computed using a random-effects model. Weights represent each study’s relative contribution to the overall pooled estimate. Confidence intervals might exceed 1.00 because standard errors have been estimated due to missing data

Seven studies reported AUC measures for classifying AD-dementia versus healthy controls (see Fig. 5A). Of these, five did not report any measures of variability; the standard error for these AUC values was therefore estimated. The pooled AUC was 0.88 [95% confidence interval (CI) 0.85–0.92] (Fig. 5A), with significant heterogeneity across studies.

#### Healthy controls versus cognitive impairment

Nine studies used ML algorithms to classify cognitive impairment versus healthy controls (see Fig. 5B). Only two of these studies reported measures of variability, therefore all other standard errors for the AUC were estimated. The pooled AUC was 0.84 [95% CI 0.74–0.95], with significant heterogeneity among studies (Fig. 5B) [32, 33, 51, 58, 79, 85, 91, 96, 98].

#### Healthy controls versus all-cause dementia

Nine studies utilised ML methods to diagnose all-cause dementia versus healthy controls, of which two studies (Chan et al. 2023 [32] and Lee et al. 2022 [47]) made multiple comparisons. Here we selected the comparisons with the largest sample sizes included. Of these studies, three reported measures of variability for the AUC, the standard error for all remaining AUC values were therefore estimated. The pooled AUC was 0.88 [95% CI 0.83–0.93] (Supplementary Fig. 1A), with significant heterogeneity across studies. Including the other two comparisons of Chan et al. 2023 [32] and Lee et al. 2022 [47] in the meta-analysis yielded similar results (Supplementary Fig. 1B).

### Risk of bias assessment

#### Diagnosis

For diagnostic studies, we conducted risk of bias assessment using the QUADAS-2 framework to establish potential biases regarding patient selection, index method bias, reference accuracy, blinding, consistency of references, participant inclusion, and method applicability (Supplementary Fig. 2A and Table 2A). Approximately 40% of the papers exhibited a low risk of bias across the aforementioned domains (flow and timing: *n* = 21; reference standard: *n* = 28; index test: *n* = 31; patient selection = 32). The patient selection domain had by far the highest risks of bias (*n* = 19), with studies either using case-control designs or failing to adequately disclose patient selection details. Concerns about method applicability in diagnostic studies were minimal, with 83% of studies having low risk of bias.

#### Prognosis

For prognostic studies, we conducted risk of bias assessment using the PROBAST framework to establish potential biases in prediction models regarding patient selection, predictor measurement, outcome measurement, and analysis and model evaluation (Supplementary Fig. 2B and Table 2B). Nine out of the 32 prognostic studies were rated as having a low risk of bias across all evaluated domains (participants: *n* = 23; predictors: *n* = 29; outcome: *n* = 26; analysis: *n* = 11). The analysis domain stood out as the most frequent source of concern, with 14 studies rated as having a high risk of bias. The primary reasons were unclear exclusion criteria from prognostic analyses and lack of clarity regarding how model overfitting and optimism in model performance were addressed. Concerns about applicability were minimal, with 94% of the studies showing low concerns.

## Discussion

This systematic review and meta-analysis summarised 75 studies examining the role of CSVD neuroimaging markers in ML-based diagnosis and prognosis of cognitive impairment and dementia. The field has grown substantially over the past two decades, with nearly 60% of studies being published in the last two years. Key findings reveal a mix of insights. Positively, most studies used datasets with balanced sex representation. Negatively, even though ML models leveraging CSVD features achieved high diagnostic performance, many lacked external validation and showed limited transparency regarding overfitting. Unexpectedly, most studies focused on AD rather than vascular dementia. Additionally, while XGBoost and neural networks are gaining traction, traditional methods like Cox regression, logistic regression, and SVM remain dominant. The following sections expand on these findings and place them within a broader context.

### Vascular neuroimaging and its contributions to neurodegeneration

Most studies on dementia diagnosis and prognosis focused on AD (23/30 diagnostic; 10/21 prognostic) rather than vascular dementia (4/30 diagnostic; 6/21 prognostic). For example, seven of the nine studies that compared all-cause dementia with healthy controls in the meta-analysis focused on AD-related dementia. These seven studies that incorporated vascular features in ML models demonstrated strong performance (AUC 0.88 [95%-CI 0.85, 0.92]). This indicates not only the benefit of including CSVD in AD diagnostic classifiers, but also reflects a growing interest in the role of CSVD and vascular neuroimaging in dementia, particularly in AD-related dementia.

The focus on AD surprised us, given the review’s emphasis on vascular neuroimaging and CSVD, though aspects discussed in the literature may help explain this trend. AD is the leading cause of dementia, and CSVD is one of the most common conditions in clinics [4]. It is therefore expected that CSVD co-occurs and interacts with AD as well as other neurodegenerative disorders [108–113]. The role of vascular risk factors in the pathogenesis and progression of AD is well established [114], extending beyond their mere coexistence [115]. Potential mechanisms include impairment of the neurovascular unit [116, 117], disruption of the glymphatic system [118], and hypoperfusion and hypoxia [116, 119]. This coexistence is now formally recognised in the most recently proposed AD diagnostic and staging criteria, where vascular biomarkers are identified as indicators of non-AD co-pathology that may accelerate symptom progression [7].

Considering vascular risk factors in the progression of neurological conditions such as vascular dementia and AD creates opportunities for prevention [116, 120]. Modifiable CSVD risk factors, including diabetes, hypertension, smoking, obesity, and high low-density lipoprotein cholesterol, can be addressed with cost-effective treatments, such as hypertensive medications and lifestyle changes [121]. These measures may reduce vascular risks and delay or prevent cognitive decline, potentially alleviating the societal burden of dementia. Recent studies in birth cohorts show a decline in cerebrovascular pathology [122], which may explain the decrease in age-adjusted dementia incidence [123–125]. Understanding and identifying CSVD features associated with AD using ML approaches may therefore support early diagnosis and risk prediction, permitting early effective preventive strategies.

### Underuse of CSVD markers beyond WMH

WMH—whether assessed through clinical visual ratings or computational methods—was by far the most consistently utilised CSVD marker, appearing in *all* structural MRI-based diagnostic and prognostic studies. While the emphasis on WMH is not inherently problematic, it represents a clear missed opportunity. The marked underrepresentation of other clinically relevant CSVD markers—such as lacunes, perivascular spaces, cerebral microbleeds, cortical superficial siderosis, and cortical cerebral microinfarcts—limits the capacity of ML models to fully capture the complexity of vascular contributions to neurodegenerative diseases. This overreliance on WMH may stem from the widespread availability of tools for its automated segmentation, combined with the lack of openly validated systems—either commercial or research-based—that offer comprehensive analysis of all neuroimaging CSVD markers [126].

### Recommendations

We have identified several limitations of the field and present corresponding recommendations, as summarised in BOX 1.

#### Reporting

Many studies did not report variances or confidence intervals for model performance, making it challenging to combine their findings in a meta-analysis. For future studies, *we strongly recommend the inclusion of confidence intervals for all performance metrics*, *following established reporting standards such as the TRIPOD + AI guideline* [127]. Careful consideration should also be given to the choice of metrics. For instance, in cases of pronounced class imbalance, reporting sensitivity and specificity alongside AUC can be crucial. Additionally, *we advocate for the full disclosure of all results*, *including those that do not achieve statistical significance*, *to enhance the completeness and unbiased representation of study findings*.

#### Generalisability

Despite achieving high accuracy metrics, the translation of predictive ML algorithms to real-world clinical settings requires extensive external validation. Despite this, our systematic review suggests that generalisability remains largely neglected, with less than 10% of the studies included in this review evaluating their models on hold-out external datasets. It is clear that datasets containing subjects with the exact pathology of interest are limited in availability. However, we also identified five additional studies that, despite having access to multiple datasets—and thus the opportunity to assess generalisability—chose to train and test their models on the pooled data instead of using separate datasets for independent validation. Many studies leveraged cross-validation, a method for evaluating model performance by dividing data into subsets, training on some and testing on others. While this approach helps estimate a model’s performance on unseen data, inherent similarities between the training and test sets mean it does not entirely eliminate the risk of overfitting. A recent study by Chekround and colleagues found that, even with adequately cross-validated algorithms, the performance of these ML models was consistently lower in external datasets [128]. *We thus recommend the validation on external datasets to properly assess the true performance of these algorithms*. An ML model, whether simple or complex, could still become “tuned” to the specific folds in the cross-validation process and fail to generalise to genuinely new data beyond the validation set.

#### From curated to real-world datasets

Neuropathological studies often show a complex constellation of brain pathologies across neurodegenerative dementias [129], which may exist to varying degrees alongside cerebrovascular disease [130]. These mixed or multiple pathologies can make it challenging for ML algorithms to accurately classify cases and predict disease progression, in turn lowering the robustness and generalisability of ML models. Hence, training ML algorithms that assess cerebrovascular disease in the presence of other neuropathological indicators, using features derived from well-characterised, multimodal datasets across neurodegenerative dementias, is highly desirable. *This underscores the importance of leveraging more representative*, *deeply phenotyped*, *real-world datasets that include diverse types of dementias*. As we move closer towards individualised, precision medicine approaches, such a strategy will optimise the translation of ML algorithms in clinical settings while ensuring their applicability across ethnically and socioeconomically diverse populations worldwide.

#### Fairness and representativeness

Many included studies used case-control designs, which, while useful for comparisons, often introduce selection bias, especially when controls are not well-matched to cases [19]. Additionally, several studies lacked clear inclusion/exclusion criteria, undermining replicability and generalisability. Six studies also failed to clearly specify diagnostic criteria, further impacting repeatability and reliability. Although there was a balanced representation of men and women in the included studies, only one study assessed sex differences in the classification of dementia [72]. It is well-established that women are at higher risk of developing dementia and WMH [131, 132]. Similarly, while ethnicity can modulate developing dementia [133], only six studies reported the race or ethnicity of their participants. The inconsistent reporting of demographic factors, such as race or ethnicity, raises concerns about diversity in dementia research, given that most dementia cohorts tend to be well educated, and of higher socioeconomic status than what would be expected based on census data [134, 135]. This has important implications for accurately predicting dementia risk, especially since race, ethnicity, socioeconomic background, and education are important factors that can modulate an individual’s cumulative risk for developing dementia [136–138]. Non-representative samples may limit the applicability of the findings to broader, more diverse populations. *We recommend that future cohort studies seek to collect and consistently report data from diverse populations*, *and that studies explore sex- and race/ethnicity-specific classifications of dementia.*

**BOX 1:** Recommendations to move toward clinically useful, machine learning methods applied to vascular neuroimaging for cognitive impairment and dementia

**Reporting**:
- Inclusion of measures of variance and/or confidence intervals for all performance metrics - Full disclosure of all results, including those that do not reach statistical significance
**Generalisability**:
- Use of external independent datasets for validation
**From curated to real-world datasets**
- Inclusion of diverse populations - Investigation of a broad spectrum of neurodegenerative diseases, including a greater focus on vascular dementia
**Fairness and representativeness**
- Exploration of sex-specific and race/ethnicity-specific contributions of vascular features for neurodegeneration

### Strengths and limitations

This work has major strengths. We performed a systematic search across nine databases to provide a comprehensive summary of the existing evidence on the application of vascular neuroimaging in ML-based diagnosis and prognosis of cognitive impairment and dementia. Our approach followed a carefully structured methodology to ensure both transparency and reproducibility. This included pre-registering our protocol and adhering to PRISMA guidelines. All papers included in the title and abstract screening, full-text screening, data extraction, and risk of bias assessment phases—during both the initial review and the re-run—were independently reviewed by multiple reviewers, with each paper evaluated by two reviewers and a third consulted to resolve any outstanding conflicts. We systematically assessed the risk of bias and the overall quality of the studies using established quality assessment tools, specifically QUADAS-2 and PROBAST, to ensure a robust evaluation of the evidence included in our review. Through these rigorous standards, we aimed to synthesise high-quality evidence that can guide future research to accelerate the integration of ML into clinical practice.

This work has three main limitations. First, a limitation of our meta-analysis is the significant variation in covariates and sample sizes across studies, which may have led to heterogeneity in the predictive models and potentially affected AUC comparability (see Supplementary data 2). Second, we did not assess the added value of neuroimaging markers of CSVD in diagnosing or predicting dementia, as only a few studies compared the performance of ML models with and without CSVD markers. Third, the risk of bias assessment tools used here were not designed for AI studies, and while recent efforts have been made, such tools are still in their infancy [127, 139]—the rationale for using accepted risk of bias tools for the tasks, QUADAS-2 and PROBAST.

### Future of ML and vascular neuroimaging

#### Neuroimaging

The majority of studies in our review relied on structural MRI data, with only a single study using computed tomography. A significant shift in this trend is unlikely in the coming years, though it is probable that new and advanced imaging methods will begin to appear more frequently in the literature. While assessing the human cerebral microvasculature in vivo using conventional imaging technologies remains challenging, advancements in MRI with higher field strengths (7T and above) as well as other imaging technologies help us get closer to detecting subtle vascular changes and visualising small-calibre blood vessels [140, 141].

The recent incorporating of non-lesional outcomes into the assessment of CSVD in the STRIVE II criteria (Standards for Reporting Vascular Changes on Neuroimaging) will likely enrich our understanding of CSVD and its contributions to neurodegenerative diseases. Investigating functional markers, such as those derived from fMRI [142], could enable determining the extent to which CSVD leads to impaired neurovascular coupling and neural network communication, and ultimately contributes to cognitive decline [143].

#### Harmonisation

A major challenge in developing large multimodal datasets lies in harmonising data. This is important because data acquired from different MRI scanners or imaging sites can introduce systematic variability—such as differences in intensity scaling, resolution, or noise characteristics—that is unrelated to the condition of interest. Without appropriate harmonisation, these scanner- or site-specific effects can confound statistical analyses, reduce model generalisability, compromise reproducibility and comparability across studies or cohorts. The fact that only a quarter (26%) of studies using data from multiple scanners or sites applied any form of harmonisation indicates that there is still considerable scope for wider adoption of such techniques to improve the reliability and translational impact of neuroimaging research.

#### Treatments

Identifying and quantifying CSVD is relevant for the selection and stratification of dementia treatments, as illustrated in anti-amyloid therapies (e.g. Lecanemab or Donanemab). As CSVD markers play a pivotal role in the diagnosis of cerebral amyloid angiopathy [144], a major risk factor for adverse treatment outcomes [144–147], accurate identification of patients who are not at increased risks of adverse events and therefore will benefit most from treatment is crucial. AI-based predictions could provide clinicians with valuable tools to tailor therapeutics, thus enhancing safety and efficacy while facilitating precision medicine approaches for patients [148].

## Conclusion

CSVD markers are playing an increasing role in ML-based diagnosis and prognosis of dementia and cognitive impairment, with models leveraging these markers already demonstrating strong performance in distinguishing individuals with and without cognitive impairment and dementia. However, challenges remain of reporting standards, generalisability, and fairness that hinder the widespread adoption of these approaches. As the cerebrovascular interest subgroup of the international DEMON Network Imaging Working Group, we have collaboratively developed a set of targeted recommendations to drive progress in this field over the next decade. We firmly believe that adopting these recommendations will greatly accelerate the integration of ML methods into clinical practice, delivering meaningful benefits for patients.

## Electronic supplementary material

Below is the link to the electronic supplementary material.

Supplementary Material 1

Supplementary Material 2

## Data Availability

Data is provided within the manuscript or supplementary information files.

## Abbreviations

AD: Alzheimer’s diseases
ADDTC: Alzheimer’s Disease Diagnostic and Treatment Centers
ADNI: Alzheimer’s Disease Neuroimaging Initiative
ADRDA: Alzheimer’s Disease and Related Disorders Association
AUC: Area under the curve
CDR: Clinical Dementia Rating
CNN: Convolutional neural network
CSVD: Cerebral small vessel diseases
DEMON: Network The Deep Dementia Phenotyping Network
DSM: Diagnostic and Statistical Manual of Mental Disorders
fMRI: Functional magnetic resonance imaging
HARNESS: HARmoNizing Brain Imaging MEthodS for VaScular Contributions to Neurodegeneration
MCI: Mild cognitive impairment
ML: Machine learning
MRI: Magnetic resonance imaging
NIA-AA: National Institute on Aging and Alzheimer’s Association
NIA-ADRC: National Institute on Aging-Alzheimer’s Disease Centers
NINCDS: National Institute of Neurological and Communicative Disorders and Stroke
NINDS-AIREN: National Institute of Neurological Disorders and Stroke and Association Internationale pour la Recherche et l’Enseignement en Neurosciences
MMSE: Mini-Mental State Examination
MoCA: Montreal Cognitive Assessment
PRESS: Peer Review of Electronic Search Strategies
PRISMA: Preferred Reporting Items for Systematic Reviews and Meta-Analysis
PROBAST: Prediction model Risk Of Bias ASsessment Tool
PROSPERO: International Prospective Register of Systematic Reviews
QUADAS-2: QUality Assessment of Diagnostic Accuracy Studies
SD: Standard deviation
STRIVE: Standards for Reporting Vascular Changes on Neuroimaging
SVM: Support vector machines
T: Tesla
TRIPOD + AI: Transparent Reporting of a multivariable prediction model for Individual Prognosis Or Diagnosis + Artificial Intelligence
USA: United States of America
WMH: White matter hyperintensities
XGBoost: eXtreme Gradient Boosting

## References

[1] Duering M, Biessels GJ, Brodtmann A, Chen C, Cordonnier C, de Leeuw F-E, et al. Neuroimaging standards for research into small vessel disease-advances since 2013. Lancet Neurol. 2023;22(7):602–18. 37236211 10.1016/S1474-4422(23)00131-X

[2] Wardlaw JM, Smith C, Dichgans M. Small vessel disease: mechanisms and clinical implications. Lancet Neurol. 2019;18(7):684–96. 31097385 10.1016/S1474-4422(19)30079-1

[3] Ter Telgte A, Duering M. Cerebral small vessel disease: advancing knowledge with neuroimaging. Stroke. 2024;55(6):1686–8. 38328947 10.1161/STROKEAHA.123.044294

[4] Cannistraro RJ, Badi M, Eidelman BH, Dickson DW, Middlebrooks EH, Meschia JF. CNS small vessel disease: A clinical review. Neurology. 2019;92(24):1146–56. 31142635 10.1212/WNL.0000000000007654 PMC6598791

[5] Clancy U, Appleton JP, Arteaga C, Doubal FN, Bath PM, Wardlaw JM. Clinical management of cerebral small vessel disease: a call for a holistic approach. Chin Med J (Engl). 2020;134(2):127–42. 33118960 10.1097/CM9.0000000000001177 PMC7817338

[6] Yang HD, Kim DH, Lee SB, Young LD. History of alzheimer’s disease. Dement Neurocogn Disord. 2016;15(4):115–21. 30906352 10.12779/dnd.2016.15.4.115 PMC6428020

[7] Jack CR Jr, Andrews JS, Beach TG, Buracchio T, Dunn B, Graf A et al. Revised criteria for diagnosis and staging of Alzheimer’s disease: Alzheimer’s Association Workgroup. Alzheimer’s & Dementia. 2024;20(8):5143-69. 10.1002/alz.13859 PMC11350039 38934362

[8] Hampel H, Elhage A, Cho M, Apostolova LG, Nicoll JAR, Atri A. Amyloid-related imaging abnormalities (ARIA): radiological, biological and clinical characteristics. Brain. 2023;146(11):4414–24. 37280110 10.1093/brain/awad188 PMC10629981

[9] ter Telgte A, van Leijsen EMC, Wiegertjes K, Klijn CJM, Tuladhar AM, de Leeuw F-E. Cerebral small vessel disease: from a focal to a global perspective. Nat Rev Neurol. 2018;14(7):387–98. 29802354 10.1038/s41582-018-0014-y

[10] Gurol ME, Sacco RL, McCullough LD. Multiple faces of cerebral small vessel diseases. Stroke. 2020;51(1):9–11. 31752615 10.1161/STROKEAHA.119.027969 PMC7590926

[11] Jiang J, Wang D, Song Y, Sachdev PS, Wen W. Computer-aided extraction of select MRI markers of cerebral small vessel disease: A systematic review. NeuroImage. 2022;261:119528. 35914668 10.1016/j.neuroimage.2022.119528

[12] Waymont JMJ, Valdés Hernández MC, Bernal J, Duarte Coello R, Brown R, Chappell FM, et al. Systematic review and meta-analysis of automated methods for quantifying enlarged perivascular spaces in the brain. NeuroImage. 2024;297:120685. 38914212 10.1016/j.neuroimage.2024.120685

[13] Hu X, Liu L, Xiong M, Lu J. Application of artificial intelligence-based magnetic resonance imaging in diagnosis of cerebral small vessel disease. CNS Neurosci Ther. 2024;30(7):e14841. 39045778 10.1111/cns.14841 PMC11267174

[14] Borchert RJ, Azevedo T, Badhwar A, Bernal J, Betts M, Bruffaerts R, et al. Artificial intelligence for diagnostic and prognostic neuroimaging in dementia: A systematic review. Alzheimers Dement. 2023;19(12):5885–904. 37563912 10.1002/alz.13412

[15] Page MJ, McKenzie JE, Bossuyt PM, Boutron I, Hoffmann TC, Mulrow CD, et al. The PRISMA 2020 statement: an updated guideline for reporting systematic reviews. BMJ. 2021;372:n71. 33782057 10.1136/bmj.n71 PMC8005924

[16] McGowan J, Sampson M, Salzwedel DM, Cogo E, Foerster V, Lefebvre C. PRESS peer review of electronic search strategies: 2015 guideline statement. J Clin Epidemiol. 2016;75:40–6. 27005575 10.1016/j.jclinepi.2016.01.021

[17] Rethlefsen ML, Kirtley S, Waffenschmidt S, Ayala AP, Moher D, Page MJ, et al. PRISMA-S: an extension to the PRISMA statement for reporting literature searches in systematic reviews. Syst Rev. 2021;10(1):39. 33499930 10.1186/s13643-020-01542-z PMC7839230

[18] Bramer WM, Giustini D, de Jonge GB, Holland L, Bekhuis T. De-duplication of database search results for systematic reviews in endnote. J Med Libr Assoc. 2016;104(3):240–3. 27366130 10.3163/1536-5050.104.3.014 PMC4915647

[19] Whiting PF, Rutjes AW, Westwood ME, Mallett S, Deeks JJ, Reitsma JB, et al. QUADAS-2: a revised tool for the quality assessment of diagnostic accuracy studies. Ann Intern Med. 2011;155(8):529–36. 22007046 10.7326/0003-4819-155-8-201110180-00009

[20] Wolff RF, Moons KGM, Riley RD, Whiting PF, Westwood M, Collins GS, et al. PROBAST: A tool to assess the risk of Bias and applicability of prediction model studies. Ann Intern Med. 2019;170(1):51–8. 30596875 10.7326/M18-1376

[21] Hanley JA, McNeil BJ. The meaning and use of the area under a receiver operating characteristic (ROC) curve. Radiology. 1982;143(1):29–36. 7063747 10.1148/radiology.143.1.7063747

[22] R Core Team. R: A Language and environment for statistical computing. Vienna, Austria: R Foundation for Statistical Computing; 2021.

[23] Viechtbauer W. Conducting meta-analyses in R with the metafor package. J Stat Softw. 2010;36(3):1–48.

[24] Aam S, Einstad MS, Munthe-Kaas R, Lydersen S, Ihle-Hansen H, Knapskog AB, et al. Post-stroke cognitive Impairment-Impact of Follow-Up time and stroke subtype on severity and cognitive profile: the Nor-COAST study. Front Neurol. 2020;11:699. 32765406 10.3389/fneur.2020.00699 PMC7379332

[25] Aamodt EB, Schellhorn T, Stage E, Sanjay AB, Logan PE, Svaldi DO et al. Predicting the emergence of major neurocognitive disorder within three months after a stroke. Front Aging Neurosci. 2021;13. 10.3389/fnagi.2021.705889 PMC8418065 34489676

[26] Altieri M, Di Piero V, Pasquini M, Gasparini M, Vanacore N, Vicenzini E, et al. Delayed poststroke dementia. Neurology. 2004;62(12):2193–7. 15210881 10.1212/01.wnl.0000130501.79012.1a

[27] Appel J, Potter E, Bhatia N, Shen Q, Zhao W, Greig MT, et al. Association of white matter hyperintensity measurements on brain MR imaging with cognitive status, medial Temporal atrophy, and cardiovascular risk factors. AJNR Am J Neuroradiol. 2009;30(10):1870–6. 19643919 10.3174/ajnr.A1693 PMC7051285

[28] Belathur Suresh M, Fischl B, Salat DH. Factors influencing accuracy of cortical thickness in the diagnosis of alzheimer’s disease. Hum Brain Mapp. 2018;39(4):1500–15. 29271096 10.1002/hbm.23922 PMC5897897

[29] Binzer M, Hammernik K, Rueckert D, Zimmer VA, editors. Long-Term cognitive outcome prediction in stroke patients using Multi-task learning on imaging and tabular data. Cham: Springer Nature Switzerland; 2022.

[30] Bordin V, Coluzzi D, Rivolta MW, Baselli G, editors. Explainable AI Points to White Matter Hyperintensities for Alzheimer’s Disease Identification: a Preliminary Study. 2022 44th Annual International Conference of the IEEE Engineering in Medicine & Biology Society (EMBC); 2022 11–15 July 2022. 10.1109/EMBC48229.2022.9871306 36086369

[31] Cajanus A, Hall A, Koikkalainen J, Solje E, Tolonen A, Urhemaa T, et al. Automatic MRI quantifying methods in Behavioral-Variant frontotemporal dementia diagnosis. Dement Geriatr Cogn Dis Extra. 2018;8(1):51–9. 29606954 10.1159/000486849 PMC5869565

[32] Chan K, Fischer C, Maralani PJ, Black SE, Moody AR, Khademi A. Alzheimer’s and vascular disease classification using regional texture biomarkers in FLAIR MRI. NeuroImage: Clin. 2023;38:103385. 36989851 10.1016/j.nicl.2023.103385 PMC10074987

[33] Chen H, Huang L, Yang D, Ye Q, Guo M, Qin R, et al. Nodal global efficiency in Front-Parietal lobe mediated periventricular white matter hyperintensity (PWMH)-Related cognitive impairment. Front Aging Neurosci. 2019;11:347. 31920627 10.3389/fnagi.2019.00347 PMC6914700

[34] Chen H, Sheng X, Qin R, Luo C, Li M, Liu R, et al. Aberrant white matter microstructure as a potential diagnostic marker in alzheimer’s disease by automated Fiber quantification. Front Neurosci. 2020;14:570123. 33071742 10.3389/fnins.2020.570123 PMC7541946

[35] Chen H, Xu J, Lv W, Hu Z, Ke Z, Qin R, et al. Altered static and dynamic functional network connectivity related to cognitive decline in individuals with white matter hyperintensities. Behav Brain Res. 2023;451:114506. 37230298 10.1016/j.bbr.2023.114506

[36] Chen Y, Sha M, Zhao X, Ma J, Ni H, Gao W, et al. Automated detection of pathologic white matter alterations in alzheimer’s disease using combined diffusivity and kurtosis method. Psychiatry Res Neuroimaging. 2017;264:35–45. 28448817 10.1016/j.pscychresns.2017.04.004

[37] Ciulli S, Citi L, Salvadori E, Valenti R, Poggesi A, Inzitari D, et al. Prediction of impaired performance in trail making test in MCI patients with small vessel disease using DTI data. IEEE J Biomed Health Inf. 2016;20(4):1026–33. 10.1109/JBHI.2016.2537808 26960231

[38] Diciotti S, Ciulli S, Ginestroni A, Salvadori E, Poggesi A, Pantoni L, et al. editors. Multimodal MRI classification in vascular mild cognitive impairment. 2015 37th Annual International Conference of the IEEE Engineering in Medicine and Biology Society (EMBC); 2015 25–29 Aug. 2015. 10.1109/EMBC.2015.7319340 26737240

[39] Dyrba M, Ewers M, Wegrzyn M, Kilimann I, Plant C, Oswald A, et al. Robust automated detection of microstructural white matter degeneration in alzheimer’s disease using machine learning classification of multicenter DTI data. PLoS ONE. 2013;8(5):e64925. 23741425 10.1371/journal.pone.0064925 PMC3669206

[40] Haller S, Bartsch A, Nguyen D, Rodriguez C, Emch J, Gold G, et al. Cerebral microhemorrhage and iron deposition in mild cognitive impairment: susceptibility-weighted MR imaging assessment. Radiology. 2010;257(3):764–73. 20923870 10.1148/radiol.10100612

[41] Han L, Liu L, Hao Y, Zhang L. Diagnosis and treatment effect of convolutional neural Network-Based magnetic resonance image features on severe stroke and mental state. Contrast Media Mol Imaging. 2021;2021(1):8947789. 34385898 10.1155/2021/8947789 PMC8328714

[42] Jokinen H, Koikkalainen J, Laakso HM, Melkas S, Nieminen T, Brander A, et al. Global burden of small vessel Disease–Related brain changes on MRI predicts cognitive and functional decline. Stroke. 2020;51(1):170–8. 31699021 10.1161/STROKEAHA.119.026170 PMC6924941

[43] Joo L, Shim WH, Suh CH, Lim SJ, Heo H, Kim WS, et al. Diagnostic performance of deep learning-based automatic white matter hyperintensity segmentation for classification of the Fazekas scale and differentiation of subcortical vascular dementia. PLoS ONE. 2022;17(9):e0274562. 36107961 10.1371/journal.pone.0274562 PMC9477348

[44] Kandiah N, Mak E, Ng A, Huang S, Au WL, Sitoh YY, et al. Cerebral white matter hyperintensity in parkinson’s disease: A major risk factor for mild cognitive impairment. Parkinsonism Relat Disord. 2013;19(7):680–3. 23623194 10.1016/j.parkreldis.2013.03.008

[45] Lai Y, Xu L, Yao L, Wu X. Discriminative analysis of non-linear brain connectivity for leukoaraiosis with resting-state fMRI. SPIE; 2015.

[46] Lambert C, Zeestraten E, Williams O, Benjamin P, Lawrence AJ, Morris RG, et al. Identifying preclinical vascular dementia in symptomatic small vessel disease using MRI. NeuroImage: Clin. 2018;19:925–38. 30003030 10.1016/j.nicl.2018.06.023 PMC6039843

[47] Lee R, Choi H, Park K-Y, Kim J-M, Seok JW. Prediction of post-stroke cognitive impairment using brain FDG PET: deep learning-based approach. Eur J Nucl Med Mol Imaging. 2022;49(4):1254–62. 34599654 10.1007/s00259-021-05556-0

[48] Li B, Zhang M, Riphagen J, Morrison Yochim K, Li B, Liu J, et al. Prediction of clinical and biomarker conformed alzheimer’s disease and mild cognitive impairment from multi-feature brain structural MRI using age-correction from a large independent lifespan sample. NeuroImage: Clin. 2020;28:102387. 32871388 10.1016/j.nicl.2020.102387 PMC7476071

[49] Li R, Lai Y, Zhang Y, Yao L, Wu X. Classification of cognitive level of patients with leukoaraiosis on the basis of linear and Non-Linear functional connectivity. Front Neurol. 2017;8. 10.3389/fneur.2017.00002 PMC5243822 28154549

[50] Liang L, Zhou P, Ye C, Yang Q, Ma T. Spatial–temporal patterns of brain disconnectome in alzheimer’s disease. Hum Brain Mapp. 2023;44(11):4272–86. 37227021 10.1002/hbm.26344 PMC10318207

[51] Lin C-J, Tu P-C, Chern C-M, Hsiao F-J, Chang F-C, Cheng H-L, et al. Connectivity features for identifying cognitive impairment in presymptomatic carotid stenosis. PLoS ONE. 2014;9(1):e85441. 24454868 10.1371/journal.pone.0085441 PMC3893296

[52] Lindemer ER, Greve DN, Fischl B, Salat DH, Gomez-Isla T. White matter abnormalities and cognition in patients with conflicting diagnoses and CSF profiles. Neurology. 2018;90(17):e1461–9. 29572277 10.1212/WNL.0000000000005353 PMC5921040

[53] Ma J, Liu F, Wang Y, Ma L, Niu Y, Wang J, et al. Frequency-dependent white-matter functional network changes associated with cognitive deficits in subcortical vascular cognitive impairment. NeuroImage: Clin. 2022;36:103245. 36451351 10.1016/j.nicl.2022.103245 PMC9668649

[54] Meng D, Hosseini AA, Simpson RJ, Shaikh Q, Tench CR, Dineen RA, et al. Lesion topography and microscopic white matter tract damage contribute to cognitive impairment in symptomatic carotid artery disease. Radiology. 2017;282(2):502–15. 27598537 10.1148/radiol.2016152685 PMC5283872

[55] Mortamais M, Reynes C, Brickman AM, Provenzano FA, Muraskin J, Portet F, et al. Spatial distribution of cerebral white matter lesions predicts progression to mild cognitive impairment and dementia. PLoS ONE. 2013;8(2):e56972. 23457645 10.1371/journal.pone.0056972 PMC3572965

[56] Oppedal K, Eftestøl T, Engan K, Beyer MK, Aarsland D. Classifying dementia using local binary patterns from different regions in magnetic resonance images. Int J Biomed Imaging. 2015;2015:572567. 25873943 10.1155/2015/572567 PMC4385607

[57] Oppedal K, Engan K, Eftestøl T, Beyer M, Aarsland D. Classifying alzheimer’s disease, lewy body dementia, and normal controls using 3D texture analysis in magnetic resonance images. Biomed Signal Process Control. 2017;33:19–29.

[58] Park G, Hong J, Duffy BA, Lee JM, Kim H. White matter hyperintensities segmentation using the ensemble U-Net with multi-scale highlighting foregrounds. NeuroImage. 2021;237:118140. 33957235 10.1016/j.neuroimage.2021.118140 PMC8382044

[59] Peters F, Villeneuve S, Belleville S. Predicting progression to dementia in elderly subjects with mild cognitive impairment using both cognitive and neuroimaging predictors. J Alzheimer’s Disease. 2014;38(2):307–18. 23963293 10.3233/JAD-130842

[60] Provenzano FA, Muraskin J, Tosto G, Narkhede A, Wasserman BT, Griffith EY, et al. White matter hyperintensities and cerebral amyloidosis: necessary and sufficient for clinical expression of alzheimer disease?? JAMA Neurol. 2013;70(4):455–61. 23420027 10.1001/jamaneurol.2013.1321 PMC4124641

[61] Qin Q, Qu J, Yin Y, Liang Y, Wang Y, Xie B, et al. Unsupervised machine learning model to predict cognitive impairment in subcortical ischemic vascular disease. Alzheimers Dement. 2023;19(8):3327–38. 36786521 10.1002/alz.12971

[62] Rabin JS, Neal TE, Nierle HE, Sikkes SAM, Buckley RF, Amariglio RE, et al. Multiple markers contribute to risk of progression from normal to mild cognitive impairment. NeuroImage: Clin. 2020;28:102400. 32919366 10.1016/j.nicl.2020.102400 PMC7491146

[63] Rosano C, Aizenstein HJ, Wu M, Newman AB, Becker JT, Lopez OL, et al. Focal atrophy and cerebrovascular disease increase dementia risk among cognitively normal older adults. J Neuroimaging. 2007;17(2):148–55. 17441836 10.1111/j.1552-6569.2007.00093.x

[64] Rosano C, Perera S, Inzitari M, Newman AB, Longstreth WT, Studenski S. Digit symbol substitution test and future clinical and subclinical disorders of cognition, mobility and mood in older adults. Age Ageing. 2016;45(5):688–95. 27496932 10.1093/ageing/afw116 PMC5027641

[65] Schouten TM, Koini M, de Vos F, Seiler S, van der Grond J, Lechner A, et al. Combining anatomical, diffusion, and resting state functional magnetic resonance imaging for individual classification of mild and moderate alzheimer’s disease. NeuroImage: Clin. 2016;11:46–51. 26909327 10.1016/j.nicl.2016.01.002 PMC4732186

[66] Smith CD, Johnson ES, Van Eldik LJ, Jicha GA, Schmitt FA, Nelson PT, et al. Peripheral (deep) but not periventricular MRI white matter hyperintensities are increased in clinical vascular dementia compared to alzheimer’s disease. Brain Behav. 2016;6(3):e00438. 26925303 10.1002/brb3.438 PMC4754499

[67] Stebbins GT, Nyenhuis DL, Wang C, Cox JL, Freels S, Bangen K, et al. Gray matter atrophy in patients with ischemic stroke with cognitive impairment. Stroke. 2008;39(3):785–93. 18258824 10.1161/STROKEAHA.107.507392

[68] Stephan BC, Tzourio C, Auriacombe S, Amieva H, Dufouil C, Alpérovitch A, et al. Usefulness of data from magnetic resonance imaging to improve prediction of dementia: population based cohort study. BMJ. 2015;350:h2863. 26099688 10.1136/bmj.h2863 PMC4476487

[69] Tang L, Wu X, Liu H, Wu F, Song R, Zhang W, et al. Individualized prediction of early alzheimer’s disease based on magnetic resonance imaging radiomics, clinical, and laboratory examinations: A 60-Month Follow-Up study. J Magn Reson Imaging. 2021;54(5):1647–57. 33987915 10.1002/jmri.27689

[70] Tozer DJ, Zeestraten E, Lawrence AJ, Barrick TR, Markus HS. Texture analysis of T1-Weighted and Fluid-Attenuated inversion recovery images detects abnormalities that correlate with cognitive decline in small vessel disease. Stroke. 2018;49(7):1656–61. 29866751 10.1161/STROKEAHA.117.019970 PMC6022812

[71] Tu MC, Huang SM, Hsu YH, Yang JJ, Lin CY, Kuo LW. Discriminating subcortical ischemic vascular disease and alzheimer’s disease by diffusion kurtosis imaging in segregated thalamic regions. Hum Brain Mapp. 2021;42(7):2018–31. 33416206 10.1002/hbm.25342 PMC8046043

[72] Twait EL, Andaur Navarro CL, Gudnason V, Hu Y-H, Launer LJ, Geerlings MI. Dementia prediction in the general population using clinically accessible variables: a proof-of-concept study using machine learning. The AGES-Reykjavik study. BMC Med Inf Decis Mak. 2023;23(1):168. 10.1186/s12911-023-02244-x PMC10463542 37641038

[73] Verdelho A, Madureira S, Ferro JM, Baezner H, Blahak C, Poggesi A, et al. Physical activity prevents progression for cognitive impairment and vascular dementia: results from the LADIS (Leukoaraiosis and Disability) study. Stroke. 2012;43(12):3331–5. 23117721 10.1161/STROKEAHA.112.661793

[74] Wan MD, Liu H, Liu XX, Zhang WW, Xiao XW, Zhang SZ, et al. Associations of multiple visual rating scales based on structural magnetic resonance imaging with disease severity and cerebrospinal fluid biomarkers in patients with alzheimer’s disease. Front Aging Neurosci. 2022;14:906519. 35966797 10.3389/fnagi.2022.906519 PMC9374170

[75] Wang J, Knol MJ, Tiulpin A, Dubost F, de Bruijne M, Vernooij MW, et al. Gray matter age prediction as a biomarker for risk of dementia. Proc Natl Acad Sci U S A. 2019;116(42):21213–8. 31575746 10.1073/pnas.1902376116 PMC6800321

[76] Wang Y, Xu C, Park JH, Lee S, Stern Y, Yoo S, et al. Diagnosis and prognosis of alzheimer’s disease using brain morphometry and white matter connectomes. Neuroimage Clin. 2019;23:101859. 31150957 10.1016/j.nicl.2019.101859 PMC6541902

[77] West NA, Windham BG, Knopman DS, Shibata DK, Coker LH, Mosley TH. Jr. Neuroimaging findings in midlife and risk of late-life dementia over 20 years of follow-up. Neurology. 2019;92(9):e917–23. 30659141 10.1212/WNL.0000000000006989 PMC6404467

[78] Williams OA, Zeestraten EA, Benjamin P, Lambert C, Lawrence AJ, Mackinnon AD, et al. Predicting dementia in cerebral small vessel disease using an automatic diffusion tensor image segmentation technique. Stroke. 2019;50(10):2775–82. 31510902 10.1161/STROKEAHA.119.025843 PMC6756294

[79] Xie Y, Cui Z, Zhang Z, Sun Y, Sheng C, Li K, et al. Identification of amnestic mild cognitive impairment using Multi-Modal brain features: A combined structural MRI and diffusion tensor imaging study. J Alzheimer’s Disease. 2015;47(2):509–22. 26401572 10.3233/JAD-150184

[80] Yao M, Zhu YC, Soumaré A, Dufouil C, Mazoyer B, Tzourio C, et al. Hippocampal perivascular spaces are related to aging and blood pressure but not to cognition. Neurobiol Aging. 2014;35(9):2118–25. 24731517 10.1016/j.neurobiolaging.2014.03.021

[81] Zhang W, Li M, Zhou X, Huang C, Wan K, Li C, et al. Altered serum amyloid beta and cerebral perfusion and their associations with cognitive function in patients with subcortical ischemic vascular disease. Front Neurosci. 2022;16:993767. 36312019 10.3389/fnins.2022.993767 PMC9608371

[82] Zhang W, Zheng X, Li R, Liu M, Xiao W, Huang L, et al. Research on nonstroke dementia screening and cognitive function prediction model for older people based on brain atrophy characteristics. Brain Behav. 2022;12(11):e2726. 36278400 10.1002/brb3.2726 PMC9660432

[83] Zhang Y, Tartaglia MC, Schuff N, Chiang GC, Ching C, Rosen HJ, et al. MRI signatures of brain macrostructural atrophy and microstructural degradation in frontotemporal Lobar degeneration subtypes. J Alzheimer’s Disease. 2013;33(2):431–44. 22976075 10.3233/JAD-2012-121156 PMC3738303

[84] Zhao J, Ding X, Du Y, Wang X, Men G. Functional connectivity between white matter and Gray matter based on fMRI for alzheimer’s disease classification. Brain Behav. 2019;9(10):e01407. 31512413 10.1002/brb3.1407 PMC6790327

[85] Zhu W, Huang H, Yang S, Luo X, Zhu W, Xu S, et al. Dysfunctional architecture underlies white matter hyperintensities with and without cognitive impairment. J Alzheimer’s Disease. 2019;71(2):461–76. 31403946 10.3233/JAD-190174

[86] Chen J, Yang J, Shen D, Wang X, Lin Z, Chen H, et al. A predictive model of the progression to alzheimer’s disease in patients with mild cognitive impairment based on the MRI enlarged perivascular spaces. J Alzheimer’s Disease. 2024;101(1):159–73. 39177602 10.3233/JAD-240523

[87] Chen Y, Lu P, Wu S, Yang J, Liu W, Zhang Z, et al. CD163-Mediated Small-Vessel injury in alzheimer’s disease: an exploration from neuroimaging to transcriptomics. Int J Mol Sci. 2024;25(4):2293. 38396970 10.3390/ijms25042293 PMC10888773

[88] Chen Y, Tozer D, Li R, Li H, Tuladhar A, De Leeuw FE, et al. Improved dementia prediction in cerebral small vessel disease using deep Learning–Derived diffusion scalar maps from T1. Stroke. 2024;55(9):2254–63. 39145386 10.1161/STROKEAHA.124.047449 PMC11346716

[89] Crystal O, Maralani PJ, Black S, Fischer C, Moody AR, Khademi A. Detecting conversion from mild cognitive impairment to alzheimer’s disease using FLAIR MRI biomarkers. Neuroimage Clin. 2023;40:103533. 37952286 10.1016/j.nicl.2023.103533 PMC10666029

[90] De Francesco S, Crema C, Archetti D, Muscio C, Reid RI, Nigri A, et al. Differential diagnosis of neurodegenerative dementias with the explainable MRI based machine learning algorithm MUQUBIA. Sci Rep. 2023;13(1):17355. 37833302 10.1038/s41598-023-43706-6 PMC10575864

[91] Feng J, Hui D, Zheng Q, Guo Y, Xia Y, Shi F, et al. Automatic detection of cognitive impairment in patients with white matter hyperintensity and causal analysis of related factors using artificial intelligence of MRI. Comput Biol Med. 2024;178:108684. 38852399 10.1016/j.compbiomed.2024.108684

[92] Keller JA, Sigurdsson S, Schmitz Abecassis B, Kant IMJ, Van Buchem MA, Launer LJ, et al. Identification of distinct brain MRI phenotypes and their association with Long-Term dementia risk in Community-Dwelling older adults. Neurology. 2024;102(7):e209176. 38471053 10.1212/WNL.0000000000209176 PMC11033985

[93] Lee M-W, Kim HW, Choe YS, Yang HS, Lee J, Lee H, et al. A multimodal machine learning model for predicting dementia conversion in alzheimer’s disease. Sci Rep. 2024;14(1):12276. 38806509 10.1038/s41598-024-60134-2 PMC11133319

[94] Li R, Harshfield EL, Bell S, Burkhart M, Tuladhar AM, Hilal S, et al. Predicting incident dementia in cerebral small vessel disease: comparison of machine learning and traditional statistical models. Cereb Circulation - Cognition Behav. 2023;5:100179. 10.1016/j.cccb.2023.100179 PMC10428032 37593075

[95] Marzi C, Scheda R, Salvadori E, Giorgio A, De Stefano N, Poggesi A et al. Fractal dimension of the cortical Gray matter outweighs other brain MRI features as a predictor of transition to dementia in patients with mild cognitive impairment and leukoaraiosis. Front Hum Neurosci. 2023;17. 10.3389/fnhum.2023.1231513 PMC10562576 37822707

[96] Shi Y, Deng J, Mao H, Han Y, Gao Q, Zeng S, et al. Macrophage migration inhibitory factor as a potential plasma biomarker of cognitive impairment in cerebral small vessel disease. ACS Omega. 2024;9(13):15339–49. 38585104 10.1021/acsomega.3c10126 PMC10993283

[97] Strain JF, Phuah C-L, Adeyemo B, Cheng K, Womack KB, McCarthy J, et al. White matter hyperintensity longitudinal morphometric analysis in association with alzheimer disease. Alzheimer’s Dement. 2023;19(10):4488–97. 37563879 10.1002/alz.13377 PMC10592317

[98] Zhu XW, Liu SB, Ji CH, Liu JJ, Huang C. Machine learning-based prediction of mild cognitive impairment among individuals with normal cognitive function. Front Neurol. 2024;15. 10.3389/fneur.2024.1352423 PMC10870793 38370526

[99] Altieri M, Di Piero V, Pasquini M, Gasparini M, Vanacore N, Vicenzini E, et al. Delayed poststroke dementia: a 4-year follow-up study. Neurology. 2004;62(12):2193–7. 15210881 10.1212/01.wnl.0000130501.79012.1a

[100] American Psychiatric Association D, American Psychiatric Association D. Diagnostic and statistical manual of mental disorders: DSM-5. American psychiatric association Washington, DC; 2013.

[101] McKhann G, Drachman D, Folstein M, Katzman R, Price D, Stadlan EM. Clinical diagnosis of alzheimer’s disease: report of the NINCDS-ADRDA work group under the auspices of department of health and human services task force on alzheimer’s disease. Neurology. 1984;34(7):939–44. 6610841 10.1212/wnl.34.7.939

[102] Jack CR Jr, Bennett DA, Blennow K, Carrillo MC, Dunn B, Haeberlein SB, et al. NIA-AA research framework: toward a biological definition of alzheimer’s disease. Alzheimer’s Dement. 2018;14(4):535–62. 29653606 10.1016/j.jalz.2018.02.018 PMC5958625

[103] Petersen RC, Wiste HJ, Weigand SD, Fields JA, Geda YE, Graff-Radford J, et al. NIA-AA alzheimer’s disease framework: clinical characterization of stages. Ann Neurol. 2021;89(6):1145–56. 33772866 10.1002/ana.26071 PMC8131266

[104] Román GC, Tatemichi TK, Erkinjuntti T, Cummings JL, Masdeu JC, Garcia JH, et al. Vascular Dement Neurol. 1993;43(2):250. 10.1212/wnl.43.2.250 8094895

[105] Chui HC, Victoroff JI, Margolin D, Jagust W, Shankle R, Katzman R. Criteria for the diagnosis of ischemic vascular dementia proposed by the state of California alzheimer’s disease diagnostic and treatment centers. Neurology. 1992;42(3):473. 1549205 10.1212/wnl.42.3.473

[106] Petersen RC, Smith GE, Waring SC, Ivnik RJ, Tangalos EG, Kokmen E. Mild cognitive impairment: clinical characterization and outcome. Arch Neurol. 1999;56(3):303–8. 10190820 10.1001/archneur.56.3.303

[107] Petersen RC. Mild cognitive impairment as a diagnostic entity. J Intern Med. 2004;256(3):183–94. 15324362 10.1111/j.1365-2796.2004.01388.x

[108] Attems J, Jellinger KA. The overlap between vascular disease and alzheimer’s disease–lessons from pathology. BMC Med. 2014;12:206. 25385447 10.1186/s12916-014-0206-2 PMC4226890

[109] Dadar M, Manera AL, Ducharme S, Collins DL. White matter hyperintensities are associated with grey matter atrophy and cognitive decline in alzheimer’s disease and frontotemporal dementia. Neurobiol Aging. 2022;111:54–63. 34968832 10.1016/j.neurobiolaging.2021.11.007

[110] Dadar M, Camicioli R, Duchesne S, Collins DL, Initiative ftAsDN. The temporal relationships between white matter hyperintensities, neurodegeneration, amyloid beta, and cognition. Alzheimer’s & Dementia, Diagnosis. Assessment & Disease Monitoring. 2020;12(1):e12091. 10.1002/dad2.12091 PMC7552231 33083512

[111] Garnier-Crussard A, Bougacha S, Wirth M, Dautricourt S, Sherif S, Landeau B, et al. White matter hyperintensity topography in alzheimer’s disease and links to cognition. Alzheimers Dement. 2022;18(3):422–33. 34322985 10.1002/alz.12410 PMC9292254

[112] Liu Y, Braidy N, Poljak A, Chan DKY, Sachdev P. Cerebral small vessel disease and the risk of alzheimer’s disease: A systematic review. Ageing Res Rev. 2018;47:41–8. 29898422 10.1016/j.arr.2018.06.002

[113] McKay E, Counts SE. Multi-Infarct dementia: A historical perspective. Dement Geriatr Cogn Dis Extra. 2017;7(1):160–71. 28626470 10.1159/000470836 PMC5471781

[114] Kivipelto M, Helkala E-L, Laakso MP, Hänninen T, Hallikainen M, Alhainen K, et al. Midlife vascular risk factors and alzheimer’s disease in later life: longitudinal, population based study. BMJ. 2001;322(7300):1447. 11408299 10.1136/bmj.322.7300.1447 PMC32306

[115] Sweeney MD, Montagne A, Sagare AP, Nation DA, Schneider LS, Chui HC, et al. Vascular dysfunction—The disregarded partner of alzheimer’s disease. Alzheimer’s Dement. 2019;15(1):158–67. 30642436 10.1016/j.jalz.2018.07.222 PMC6338083

[116] Huang W, Xia Q, Zheng F, Zhao X, Ge F, Xiao J, et al. Microglia-Mediated neurovascular unit dysfunction in alzheimer’s disease. J Alzheimers Dis. 2023;94(s1):S335–54. 36683511 10.3233/JAD-221064 PMC10473143

[117] Zimmerman B, Rypma B, Gratton G, Fabiani M. Age-related changes in cerebrovascular health and their effects on neural function and cognition: A comprehensive review. Psychophysiology. 2021;58(7):e13796. 33728712 10.1111/psyp.13796 PMC8244108

[118] Nedergaard M, Goldman SA. Glymphatic failure as a final common pathway to dementia. Science. 2020;370(6512):50–6. 33004510 10.1126/science.abb8739 PMC8186542

[119] Laing KK, Simoes S, Baena-Caldas GP, Lao PJ, Kothiya M, Igwe KC, et al. Cerebrovascular disease promotes Tau pathology in alzheimer’s disease. Brain Commun. 2020;2(2):fcaa132. 33215083 10.1093/braincomms/fcaa132 PMC7660042

[120] Backhouse EV, Boardman JP, Wardlaw JM. Cerebral small vessel disease: Early-Life antecedents and Long-Term implications for the brain, aging, stroke, and dementia. Hypertension. 2024;81(1):54–74. 37732415 10.1161/HYPERTENSIONAHA.122.19940 PMC10734792

[121] Livingston G, Huntley J, Liu KY, Costafreda SG, Selbæk G, Alladi S, et al. Dementia prevention, intervention, and care: 2024 report of the < em > lancet standing commission. Lancet. 2024;404(10452):572–628. 39096926 10.1016/S0140-6736(24)01296-0

[122] Grodstein F, Leurgans SE, Capuano AW, Schneider JA, Bennett DA. Trends in postmortem neurodegenerative and cerebrovascular neuropathologies over 25 years. JAMA Neurol. 2023;80(4):370–6. 36805154 10.1001/jamaneurol.2022.5416 PMC9941972

[123] Satizabal CL, Beiser AS, Chouraki V, Chêne G, Dufouil C, Seshadri S. Incidence of dementia over three decades in the Framingham heart study. N Engl J Med. 2016;374(6):523–32. 26863354 10.1056/NEJMoa1504327 PMC4943081

[124] Derby CA, Katz MJ, Lipton RB, Hall CB. Trends in dementia incidence in a birth cohort analysis of the Einstein aging study. JAMA Neurol. 2017;74(11):1345–51. 28873124 10.1001/jamaneurol.2017.1964 PMC5710583

[125] Tom SE, Phadke M, Hubbard RA, Crane PK, Stern Y, Larson EB. Association of demographic and Early-Life socioeconomic factors by birth cohort with dementia incidence among US adults born between 1893 and 1949. JAMA Netw Open. 2020;3(7):e2011094–e. 32716513 10.1001/jamanetworkopen.2020.11094 PMC8794045

[126] Phitidis J, O’Neil AQ, Whiteley WN, Alex B, Wardlaw JM, Bernabeu MO, et al. Automated neuroradiological support systems for multiple cerebrovascular disease markers — A systematic review and meta-analysis. Comput Methods Programs Biomed. 2025;264:108715. 40096783 10.1016/j.cmpb.2025.108715

[127] Collins GS, Moons KGM, Dhiman P, Riley RD, Beam AL, Van Calster B, et al. TRIPOD + AI statement: updated guidance for reporting clinical prediction models that use regression or machine learning methods. BMJ. 2024;385:e078378. 38626948 10.1136/bmj-2023-078378 PMC11019967

[128] Chekroud AM, Hawrilenko M, Loho H, Bondar J, Gueorguieva R, Hasan A, et al. Illusory generalizability of clinical prediction models. Science. 2024;383(6679):164–7. 38207039 10.1126/science.adg8538

[129] Robinson JL, Xie SX, Baer DR, Suh E, Van Deerlin VM, Loh NJ, et al. Pathological combinations in neurodegenerative disease are heterogeneous and disease-associated. Brain. 2023;146(6):2557–69. 36864661 10.1093/brain/awad059 PMC10232273

[130] Rahimi J, Kovacs GG. Prevalence of mixed pathologies in the aging brain. Alzheimers Res Ther. 2014;6(9):82. 25419243 10.1186/s13195-014-0082-1 PMC4239398

[131] Mielke MM. Sex and gender differences in alzheimer’s disease dementia. Psychiatr Times. 2018;35(11):14–7. 30820070 PMC6390276

[132] Lohner V, Pehlivan G, Sanroma G, Miloschewski A, Schirmer MD, Stöcker T, et al. Relation between sex, menopause, and white matter hyperintensities: the Rhineland study. Neurology. 2022;99(9):e935–43. 35768207 10.1212/WNL.0000000000200782 PMC9502737

[133] Shiekh SI, Cadogan SL, Lin LY, Mathur R, Smeeth L, Warren-Gash C. Ethnic differences in dementia risk: A systematic review and Meta-Analysis. J Alzheimers Dis. 2021;80(1):337–55. 33554910 10.3233/JAD-201209 PMC8075390

[134] Demarest S, Van der Heyden J, Charafeddine R, Tafforeau J, Van Oyen H, Van Hal G. Socio-economic differences in participation of households in a Belgian National health survey. Eur J Public Health. 2013;23(6):981–5. 23183496 10.1093/eurpub/cks158

[135] Korkeila K, Suominen S, Ahvenainen J, Ojanlatva A, Rautava P, Helenius H, et al. Non-response and related factors in a nation-wide health survey. Eur J Epidemiol. 2001;17(11):991–9. 12380710 10.1023/a:1020016922473

[136] Mayeda ER, Glymour MM, Quesenberry CP, Whitmer RA. Inequalities in dementia incidence between six Racial and ethnic groups over 14 years. Alzheimer’s Dement. 2016;12(3):216–24. 26874595 10.1016/j.jalz.2015.12.007 PMC4969071

[137] Hudomiet P, Hurd MD, Rohwedder S. Trends in inequalities in the prevalence of dementia in the united States. Proc Natl Acad Sci. 2022;119(46):e2212205119. 36343247 10.1073/pnas.2212205119 PMC9674270

[138] Deckers K, Cadar D, van Boxtel MPJ, Verhey FRJ, Steptoe A, Köhler S. Modifiable risk factors explain socioeconomic inequalities in dementia risk: evidence from a Population-Based prospective cohort study. J Alzheimer’s Dis. 2019;71:549–57. 31424404 10.3233/JAD-190541 PMC6839472

[139] Sounderajah V, Ashrafian H, Rose S, Shah NH, Ghassemi M, Golub R, et al. A quality assessment tool for artificial intelligence-centered diagnostic test accuracy studies: QUADAS-AI. Nat Med. 2021;27(10):1663–5. 34635854 10.1038/s41591-021-01517-0

[140] Osuafor CN, Rua C, Mackinnon AD, Egle M, Benjamin P, Tozer DJ, et al. Visualisation of lenticulostriate arteries using contrast-enhanced time-of-flight magnetic resonance angiography at 7 Tesla. Sci Rep. 2022;12(1):20306. 36434036 10.1038/s41598-022-24832-z PMC9700841

[141] Zwartbol MH, van der Kolk AG, Kuijf HJ, Witkamp TD, Ghaznawi R, Hendrikse J, et al. Intracranial vessel wall lesions on 7T MRI and MRI features of cerebral small vessel disease: the SMART-MR study. J Cereb Blood Flow Metabolism. 2021;41(6):1219–28. 10.1177/0271678X20958517 PMC8138333 33023386

[142] Hillman EM. Coupling mechanism and significance of the BOLD signal: a status report. Annu Rev Neurosci. 2014;37:161–81. 25032494 10.1146/annurev-neuro-071013-014111 PMC4147398

[143] Li K, Fu Z, Luo X, Zeng Q, Huang P, Zhang M, et al. The influence of cerebral small vessel disease on static and dynamic functional network connectivity in subjects along alzheimer’s disease continuum. Brain Connect. 2021;11(3):189–200. 33198482 10.1089/brain.2020.0819 PMC8080908

[144] Charidimou A, Boulouis G, Frosch MP, Baron JC, Pasi M, Albucher JF, et al. The Boston criteria version 2.0 for cerebral amyloid angiopathy: a multicentre, retrospective, MRI-neuropathology diagnostic accuracy study. Lancet Neurol. 2022;21(8):714–25. 35841910 10.1016/S1474-4422(22)00208-3 PMC9389452

[145] Sin M-K, Zamrini E, Ahmed A, Nho K, Hajjar I. Anti-Amyloid therapy, AD, and ARIA: untangling the role of CAA. J Clin Med. 2023;12(21):6792. 37959255 10.3390/jcm12216792 PMC10647766

[146] Beach TG, Sue LI, Scott S, Intorcia AJ, Walker JE, Arce RA, et al. Cerebral white matter rarefaction has both neurodegenerative and vascular causes and May primarily be a distal axonopathy. J Neuropathol Exp Neurol. 2023;82(6):457–66. 37071794 10.1093/jnen/nlad026 PMC10209646

[147] Sperling R, Salloway S, Brooks DJ, Tampieri D, Barakos J, Fox NC, et al. Amyloid-related imaging abnormalities in patients with alzheimer’s disease treated with bapineuzumab: a retrospective analysis. Lancet Neurol. 2012;11(3):241–9. 22305802 10.1016/S1474-4422(12)70015-7 PMC4063417

[148] Bates DW, Levine D, Syrowatka A, Kuznetsova M, Craig KJT, Rui A, et al. The potential of artificial intelligence to improve patient safety: a scoping review. Npj Digit Med. 2021;4(1):54. 33742085 10.1038/s41746-021-00423-6 PMC7979747

